# Removal of Oxyanions and Trace Metals from River Water Samples Using Magnetic Biopolymer/Halloysite Nanocomposites

**DOI:** 10.3390/molecules30183777

**Published:** 2025-09-17

**Authors:** Nyeleti Bridget Mabaso, Philiswa Nosizo Nomngongo, Luthando Nyaba

**Affiliations:** 1 Department of Chemical Sciences, University of Johannesburg, Doornfontein Campus, Johannesburg 2028, South Africa; 201418701@student.uj.ac.za (N.B.M.); pnnomngongo@uj.ac.za (P.N.N.); 2 Department of Science and Innovation-National Research Foundation South African Research Chair Initiative (DSI-NRF SARChI): Nanotechnology for Water, University of Johannesburg, Doornfontein 2028, South Africa

**Keywords:** adsorptive removal, chitosan, halloysite nanocomposite, magnetic biopolymers, oxyanions, river water, sodium alginate, trace metals

## Abstract

The presence of metallic pollutants presents a significant risk to human health, making their removal crucial. Magnetic halloysite nanotube (HNT@Fe_3_O_4_) nanocomposite was synthesised via co-precipitation, and then magnetic hydrogel (Fe_3_O_4_@HNT-SA and Fe_3_O_4_@HNT-CTS) nanocomposites were prepared using chitosan (CTS) and sodium alginate (SA) biopolymers. The structural, morphological, crystalline, surface, and thermal properties of the hydrogels were determined. The favourable adsorption performance of Fe_3_O_4_@HNT-SA and Fe_3_O_4_@HNT-CTS hydrogels towards As, Cd, Cr, Mo, Pb, Sb and V was established by optimising the factors affecting the sorption process. The results indicated that Fe_3_O_4_@HNT-CTS was suitable for the adsorption of As, Cr, Mo, Sb and V, while Fe_3_O_4_@HNT-SA had high adsorption affinity for Cd and Pb. The data for the adsorption of target analytes onto the hydrogels were mostly explained by both the Langmuir isotherm model and the pseudo-second order model. The maximum adsorption capacities of Fe_3_O_4_@HNT-SA hydrogel for Cd and Pb were 52.2 mg/g and 57.7 mg/g, respectively. On the other hand, the maximum capacities of the Fe_3_O_4_@HNT-CTS hydrogel for As, Cr, Mo, Sb, and V were 30.3 mg/g, 28.4 mg/g, 22.2 mg/g, 24.7 mg/g, and 19.9 mg/g, respectively. The Fe_3_O_4_@HNT-SA and Fe_3_O_4_@HNT-CTS hydrogels effectively removed the respective target analytes from river water samples.

## 1. Introduction

The introduction of metal/metalloid contaminants poses significant risks to both human and ecological health. These elements concentrate due to human activities, such as industrial operations, mining, and agricultural practices, leading to water contamination [1]. Elements such as As, Cr, Mo, Sb, Se and V, collectively known as oxyanions, are highly water-soluble, increasing the potential for accumulation in vulnerable organisms and ecological systems, and they lead to serious public health effects [2]. While trace metals, such as Cd and Pb, are recognised for their toxicity and carcinogenic properties [3]. Therefore, developing a method to remove these metals and metalloids from the environment is essential.

Some of the methods used for removing metals include chemical precipitation [4], ionic exchange [5], reversed osmosis [6], and adsorption [7]. Adsorption is regarded as one of the most effective methods for removing metallic pollutants, offering simple operation, high efficiency, and low cost [8]. As a result, it is commonly used to treat metallic pollutants in water. Metal removal efficiency depends on the properties of the adsorbent, including regenerability, accessibility, surface area and adsorption capacity [9]. Various adsorbents are employed to remove metallic contaminants from aqueous solutions. These include Zr-MOF [10], ZnO-GO-NiO [11], biochars [12], GO-Fe_3_O_4_ [13], nanobentonite [14], and MnO_2_ [15]. However, many of these adsorbents present challenges such as high costs, toxicity, low surface area, and difficulties in regeneration. Natural clays and biopolymers are particularly interesting due to their availability, low cost, and lower toxicity than other materials [16].

Sodium alginate (SA) is a naturally occurring biopolymer extracted from brown seaweed, composed of β-D-mannuronic acid and guluronic acid as its monomeric components, as detailed by [17]. It is characterised by its carboxyl and hydroxyl functional groups, which offer distinct adsorption properties, alongside its notable biocompatibility and biodegradability, as highlighted by [18]. Due to its renewable nature and availability at an economical price point, alginate presents a sustainable option. It can undergo modifications, oxidation, and esterification to enhance its properties. Nonetheless, its application is somewhat hindered by its poor mechanical and chemical stability [19].

Whereas chitosan (CTS), a polymer obtained by deacetylating chitin present in the shells of crustaceans, is distinguished by its unique qualities, making it invaluable in various fields [20]. Its ability to remove heavy metals via ion exchange and binding processes is due to its structure’s amino and hydroxyl functional groups [21]. Its appeal is further brought out by its affordability and its low-toxicity nature [22]. Nevertheless, it is prone to breakdown in acidic settings, poor solubility, and a minimal surface area limit its broader usage [23]. Various modifications of chitosan have been investigated to overcome these challenges. These include modifying metal–organic frameworks with chitosan and citric acid for Cr (VI) removal [24]. Chitosan, a polyelectrolyte, together with carboxymethylcellulose, was synthesised to eliminate dyes and heavy metals from water [25].

Furthermore, incorporating halloysite nanotubes (HNT) into CTS and SA enhances their mechanical properties and thermal stability while adding new functionalities. The tubular structure of HNTs will capture and hold ions inside and on their surface, which is beneficial for ion adsorption. Thus, this research seeks to create Fe_3_O_4_@HNT-SA and Fe_3_O_4_@HNT-CTS hydrogel nanocomposites for As, Cd, Cr, Mo, Pb, Sb and V adsorption. The central composite design was used to determine the key factors influencing the adsorption efficiency of the hydrogels. The incorporation of CTS and SA to Fe_3_O_4_@HNT revealed the change in surface characteristics of the magnetic hydrogels that led to different adsorption affinities toward the target analytes. Adsorption kinetic and isotherm models, such as Langmuir, Freundlich, pseudo-first order and pseudo-second order, were used to elucidate the adsorption mechanism. Furthermore, the possibility of using Fe_3_O_4_@HNT-SA and Fe_3_O_4_@HNT-CTS in the removal of As, Cd, Cr, Mo, Pb, Sb and V from river water was investigated.

## 2. Results and Discussion

### 2.1. Characterisation of the Hydrogel Materials

#### 2.1.1. Fourier-Transform Infrared Spectroscopy (FTIR)

**Figure 1:**
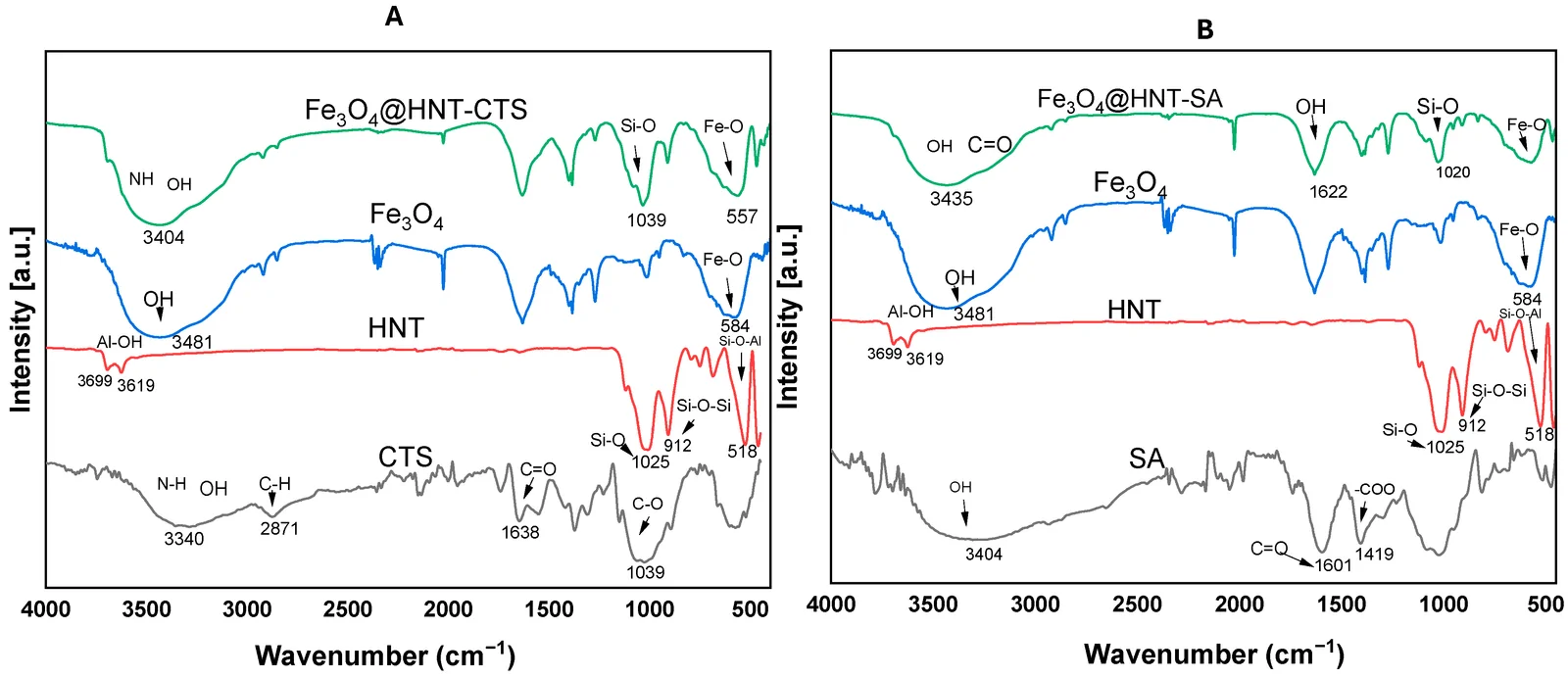
FTIR spectra of (**A**) Fe_3_O_4_@HNT-CTS (**B**) Fe_3_O_4_@HNT-SA.

Figure 1 shows FTIR spectra of SA, HNT, CTS, Fe_3_O_4_, Fe_3_O_4_@HNT-SA and Fe_3_O_4_@HNT-CTS. In the spectrum of HNT represented by Figure 1, the peaks at 3699 and 3619 cm^−1^ resulted from the internal aluminium surface (Al-OH). The peak of Si-O is at 1033 and 1025 cm^−1^ for HNT. The peaks at 912 and 914 cm^−1^ could be attributed to silica, corresponding to the asymmetrical stretching vibration of Si-O-Si. Peaks at 518 cm^−1^ correspond to Si-O-Al. The peaks of HNT identified are similar to the peaks identified by [26]. The FTIR spectrum of CTS is represented in Figure 1A. The peaks at 3093–3345 cm^−1^ correspond to N-H and O-H stretching. The peak at 2871 cm^−1^ can be attributed to C-H asymmetry; similar peaks of CTS were also observed by [27]. The 1638 and 1039 cm^−1^ peaks are attributed to -CO and C-O; they were also observed by [28]. SA spectrum is indicated in Figure 1B. The broad peak at 3404 cm^−1^ corresponds to stretching vibrations of hydroxyl (-OH) groups, the peak at 1601 cm^−1^ corresponds to C=O, and the peak at 1419 cm^−1^ corresponds to the symmetric stretching of the carboxylate groups (-COO^−^), as observed also by [29]. The Fe_3_O_4_ spectrum is represented in Figure 1. The peak at 584 cm^−1^ is attributed to Fe-O, while the peak at 3481 cm^−1^ is attributed to -OH. Figure 1A represents the spectrum of Fe_3_O_4_@HNT-CTS. In this spectrum, there is a peak at 3470 cm^−1^ due to the –OH/-NH stretch. An overlap in -OH/-NH stretching vibrations with HNT’s Si-O stretch indicates interaction between the two materials, and there are shifts in the typical band positions due to hydrogen bonding between HNT, Fe_3_O_4_, and CTS. The peak at 565 cm^−1^ results from Fe-O bond stretching, which results from Fe_3_O_4_ incorporation. Si-O is at 1044 cm^−1^, confirming the presence of HNT in the Fe_3_O_4_@HNT-CTS composite. The peak at 1643 cm^−1^ may be due to OH from Fe_3_O_4_. The spectrum of Fe_3_O_4_@HNT-SA is represented in Figure 1B. The presence of SA in combination with HNT likely shifts or overlaps the hydroxyl and carbonyl bands; this phenomenon can be seen at 3272–3711 cm^−1^. The peak at 1622 cm^−1^ (C=O stretching) overlapped with HNT peaks and OH from Fe_3_O_4_. The peak at 1032 cm^−1^ may be attributed to Si-O of HNT. The peak at 585 cm^−1^ may be attributed to the Fe-O bond stretching of Fe_3_O_4_, and the presence of peaks from Fe_3_O_4_, SA, and HNT confirms the nanocomposite formation.

#### 2.1.2. Transmission Electron Microscopy (TEM)

**Figure 2:**
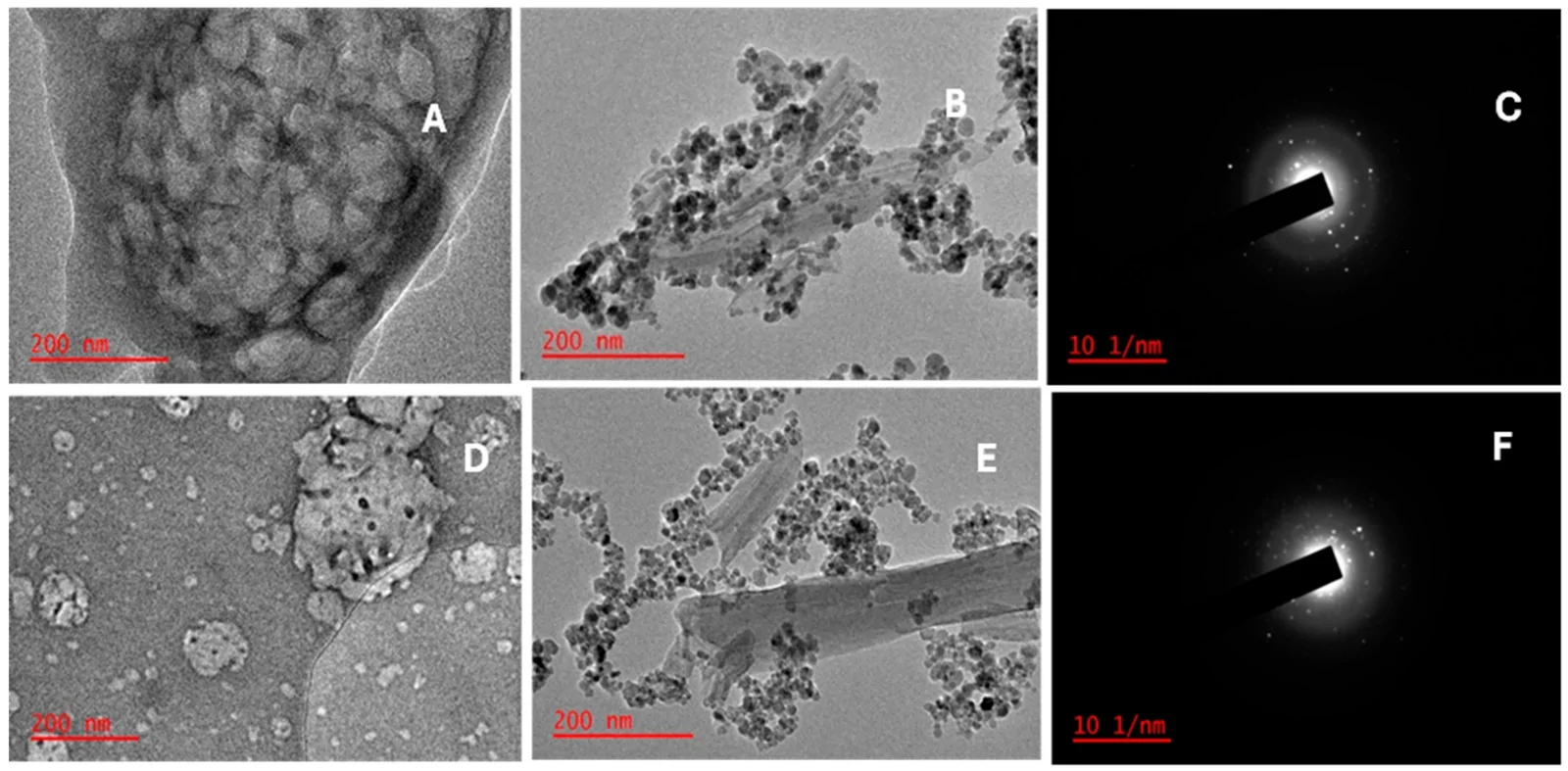
TEM images of (**A**) CTS (**B**) Fe_3_O_4_@HNT-CTS (**C**) Fe_3_O_4_@HNT-CTS (**D**) SA (**E**) SA-HNT@Fe_3_O_4_ (**F**) SA-HNT@Fe_3_O_4_.

Figure 2 shows the TEM images of SA, CTS, Fe_3_O_4_@HNT-CTS and Fe_3_O_4_@HNT-SA. Pure HNT, indicated by Figure S1, has a hollow, tubular shape, with tubes overlapping each other. while Figure S1B shows a spherical shape of magnetite. Figure 2A shows that CTS particles were clumped together. The nanocomposite Fe_3_O_4_@HNT-CTS in Figure 2B shows that the magnetic nanoparticles were attached to the exterior and the interior of HNT. Figure 2C reveals the selected area of electron diffraction of Fe_3_O_4_@HNT-CTS; the figure shows six rings corresponding to Miller indexes of (220), (311), (400), (422), (511) and (440), which agree with the XRD results and suggest that the nanocomposite was coated with Fe_3_O_4_. SA is shown in Figure 2D, which shows an irregularly shaped porous material. In the case of Fe_3_O_4_@HNT-SA demonstrated in Figure 2E, it is observed that the HNT is coated with Fe_3_O_4_, with most of the magnetic nanoparticles on the side of the tube forming a chain-like structure. Furthermore, some magnetic nanoparticles form inside the HNT. Figure 2F for SA-HNT@Fe_3_O_4_, the six rings correspond to Miller indexes (220), (311), (400), (422), (511) and (440), which also agrees with the XRD results that suggest the coating with Fe_3_O_4_.

#### 2.1.3. Scanning Electron Microscopy (SEM)

**Figure 3:**
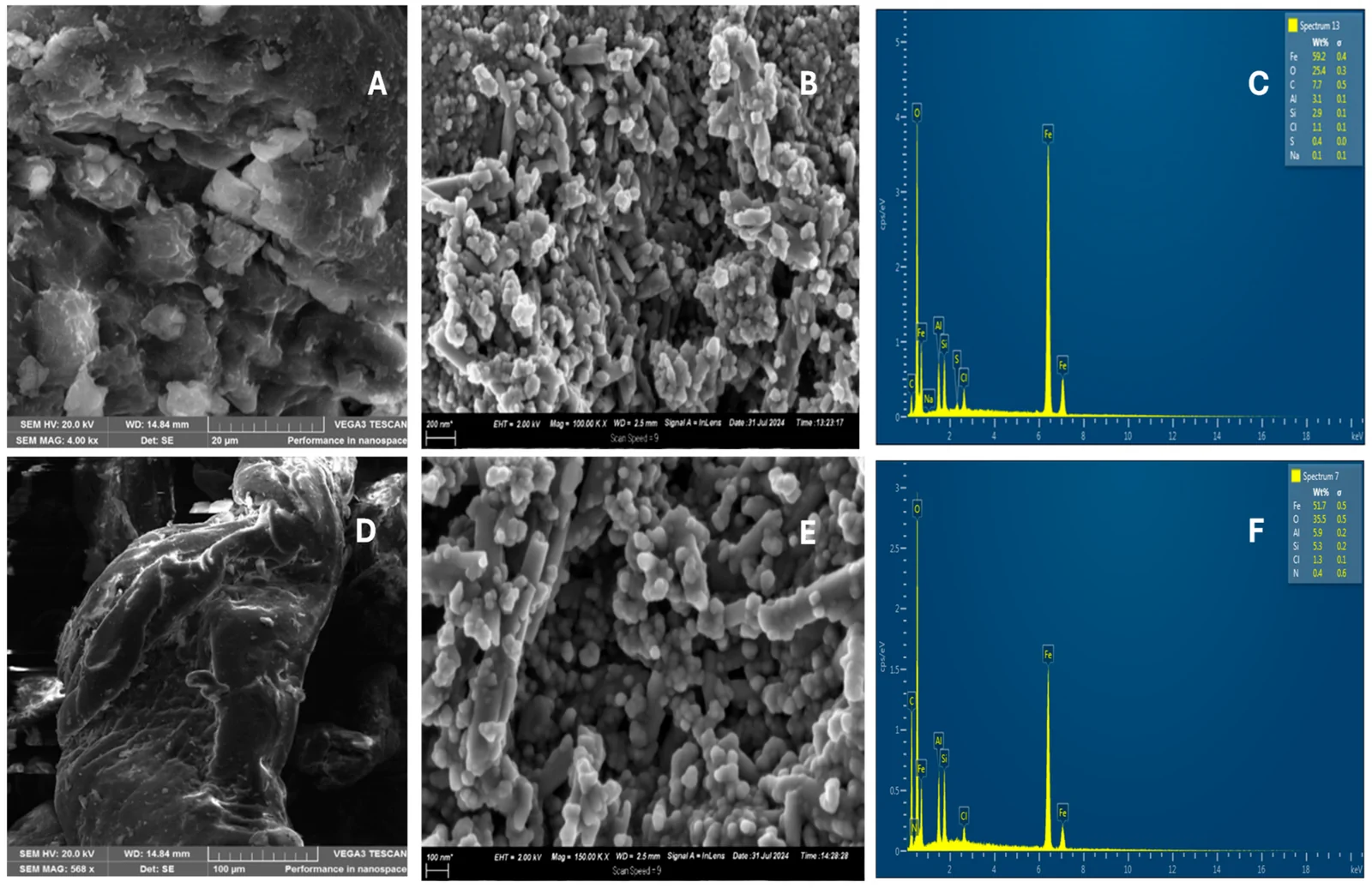
SEM (**A**) SA (**B**) Fe_3_O_4_@HNT-SA (**C**) Fe_3_O_4_@HNT-SA (EDS) (**D**) CTS (**E**) Fe_3_O_4_@HNT-CTS (**F**) Fe_3_O_4_@HNT-CTS (EDS).

Figure 3 shows the morphology of SA, CTS, Fe_3_O_4_@HNT-CTS and Fe_3_O_4_@HNT-SA, whereas the SEM images of HNT and Fe_3_O_4_ are displayed in Figure S2A,B. Figure 3A shows the morphology of SA. The nanocomposite of Fe_3_O_4_@HNT-SA shown in Figure 3B shows the spherical shape of SA and Fe_3_O_4_, which were embedded in the HNT tube. EDS of SA-HNT@Fe_3_O_4_ shown in Figure 3C reveals the presence of elements such as Si, Al, Fe, Cl, O, and C, corresponding to the elemental composition of HNT, SA and Fe_3_O_4_ precursors, thereby confirming the synthesis of the nanocomposite. Figure 3D shows the morphology of CTS. The SEM image of Fe_3_O_4_@HNT-CTS, as represented in Figure 3E, shows the nanotubes coated with spherical nanoparticles of CTS and Fe_3_O_4_. In Figure 3F, the EDS analysis of Fe_3_O_4_@HNT-CTS identifies the elemental compositions of Fe, Al, Si, N, and C from CTS, Fe_3_O_4_, and HNT, further confirming the formation of the composite.

#### 2.1.4. Brunauer–Emmett–Teller (BET)

**Figure 4:**
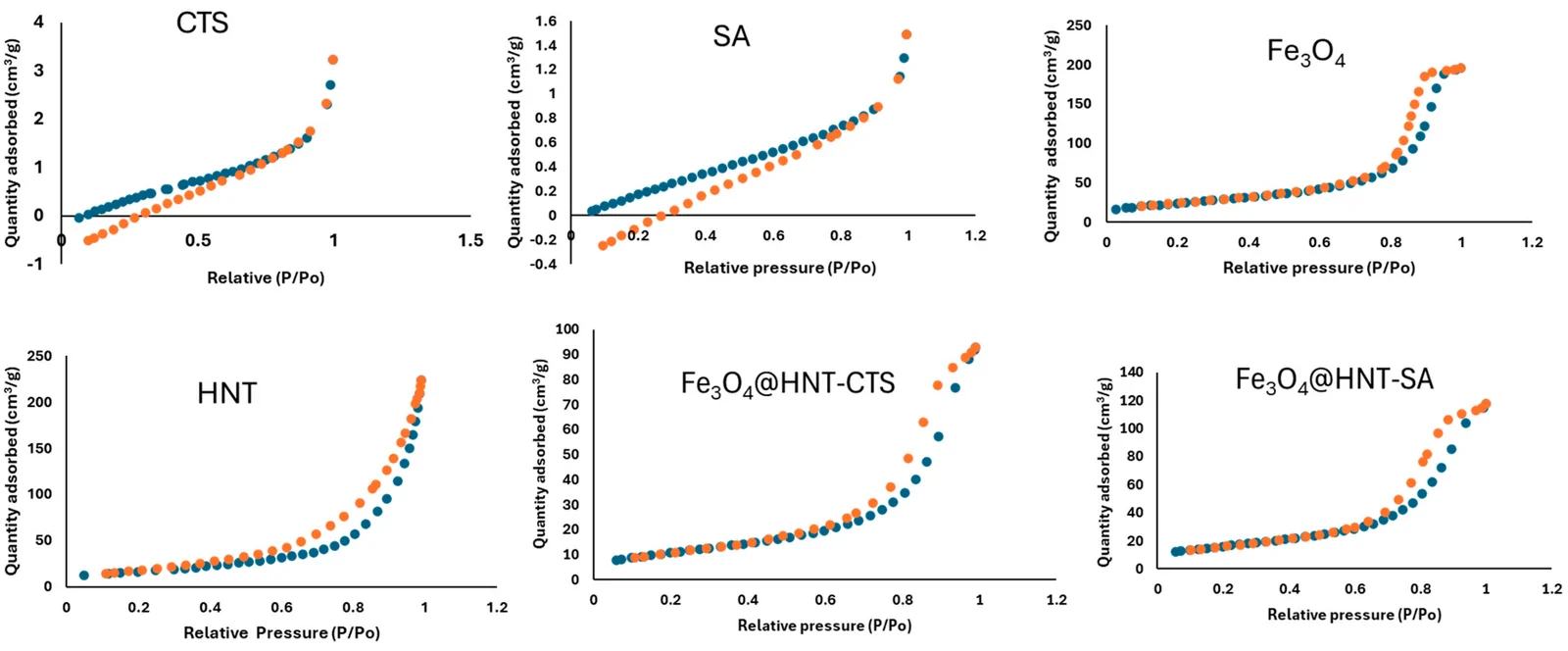
BET adsorption–desorption of isotherms of CTS, SA, Fe_3_O_4_, HNT, Fe_3_O_4_@HNT-CTS and Fe_3_O_4_@HNT-SA.

The N_2_ adsorption–desorption analysis was used to assess the surface area, pore volume, pore size distribution, and average pore size of SA, HNT, CTS, Fe_3_O_4_, Fe_3_O_4_@HNT-SA and Fe_3_O_4_@HNT-CTS. Figure 4 shows that the materials HNT exhibit type Iva with an H3 hysteresis loop, whereas Fe_3_O_4_, CTS, Fe_3_O_4_@HNT-SA and Fe_3_O_4_@HNT-CTS exhibit type Iva and H1 hysteresis loops [30], which are characteristics of the presence of mesopores. Fe_3_O_4_, at the same time, is an H1 loop. The surface area of the polymers without modification is small due to their polymeric nature (for CTS = 1.60, SA = 3.82), as shown in Table 1. Incorporating these polymers with HNT and Fe_3_O_4_ brings additional surface area and functional groups. The surface area of the nanocomposite was (Fe_3_O_4_@HNT-CTS = 58, Fe_3_O_4_@HNT-SA = 41). Also, the pore volume of Fe_3_O_4_@HNT-SA and Fe_3_O_4_@HNT-CTS increased while the average pore size decreased due to the formation of new pores.

#### 2.1.5. X-Ray Diffraction Analysis (XRD)

**Figure 5:**
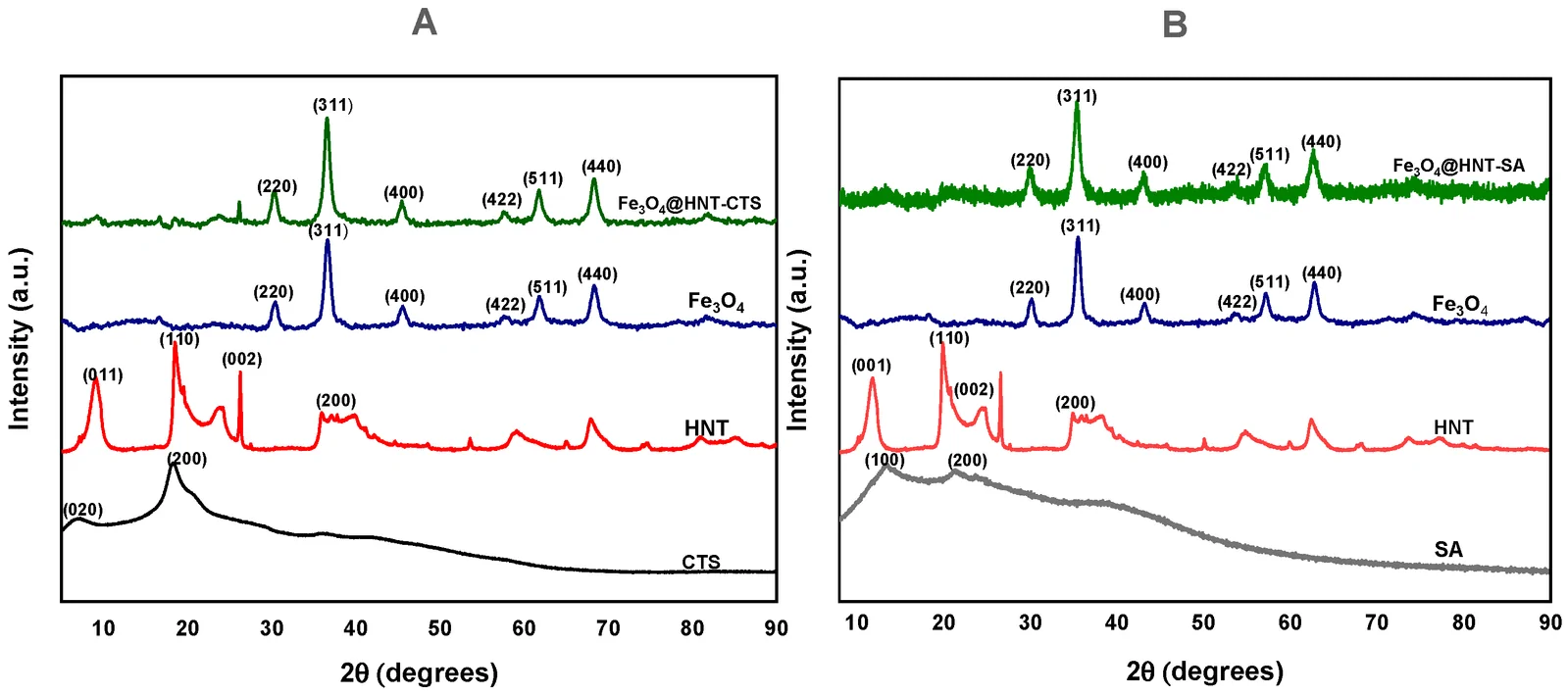
The X-Ray diffractograms (**A**) Fe_3_O_4_@HNT-CTS (**B**) Fe_3_O_4_@HNT-SA.

X-Ray diffraction analysis (XRD) was used to identify HNT, CTS, Fe_3_O_4_, SA, Fe_3_O_4_@HNT-SA and Fe_3_O_4_@HNT-CTS nanocrystalline structures. It is evident from Figure 5A,B that the diffractogram for HNTs shows reflection peaks at various diffraction angles, including 11.89°, 19.82 °, 24.87 °, 36.54°, and 38.04°, which were indexed to 001, 110, 002, 200, and 131, respectively, agreeing with what was reported by [31]. The diffractogram of Fe_3_O_4_ in Figure 5A,B has peaks including 29.69°, 34.87°, 42.47°, 56,47°, and 62.96°, which are indexed to (220), (311), (400), (422), (511) and (440), respectively, and correspond with what was reported by [32]. The peak characteristics of CTS, as indicated by Figure 5A at 2θ, were 4.3°,10.16° (020), and 19.88° (200), which were consistent with the literature [33]. The characteristic peaks of SA, as shown in Figure 5B, were 13.25° and 23.62°, which are indexed to (100) and (200), respectively; similar peaks were observed by [34]. The diffractogram of Fe_3_O_4_@HNT-CTS is shown in Figure 5A. The spectra show the peaks at 30.03°, 43.07°,57.38° and 63.06° resulting from Fe_3_O_4_ with a slight shift and the peak at 36.54° of HNT was overlapped by the Fe_3_O_4_ peak at 36.54°, and this confirms successful incorporation. There was a peak at 26.49°, which confirms the presence of HNT in the Fe_3_O_4_@HNT-CTS. The diffractogram of Fe_3_O_4_@HNT-SA is displayed in Figure 5B. The figure illustrates that Fe_3_O_4_ overlapped with the weaker peaks from SA or HNT, especially at similar 2θ values, confirming the bonding between Fe_3_O_4_ and SA and HNT.

#### 2.1.6. Zeta Potential

**Figure 6:**
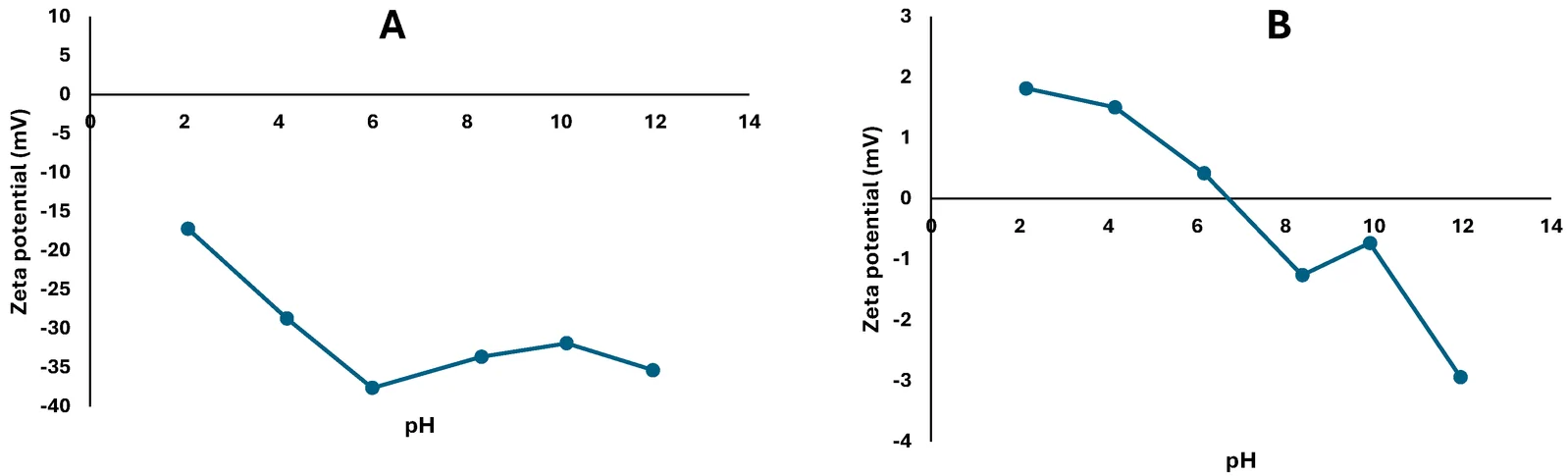
(**A**) Surface charge of Fe_3_O_4_@HNT-SA (**B**) Surface charge of Fe_3_O_4_@HNT-CTS.

The point of zero charge (pHpzc) was used to determine the surface charge of the adsorbent, which influences the adsorption of Cd and Pb on Fe_3_O_4_@HNT-SA and the adsorption of As, Cr, Mo, Sb and V using Fe_3_O_4_@HNT-CTS. As shown in Figure 6A, the zeta potential of Fe_3_O_4_@HNT-SA indicates that the adsorbent remains negatively charged across a range of pH levels. This behaviour is associated with the deprotonation of hydroxyl and carboxyl groups in Fe_3_O_4_@HNT-SA. The deprotonation increases as the pH increases, resulting in a higher concentration of negatively charged carboxylate ions and hydroxyl groups, which leads to an overall negative zeta potential for Fe_3_O_4_@HNT-SA. Figure 6B shows that the zeta potential decreased with the increase in the pH of the solution. The zeta potential was positive at 2–6, and the point of zero charge was at a pH of 7. At lower pH, the protonation of NH_2_ and hydroxyl groups results in the charge of Fe_3_O_4_@HNT-CTS becoming positive [35]. These amine groups lose protons at a higher pH above 7, producing a neutral and negative charge.

### 2.2. Adsorbent Selection

**Figure 7:**
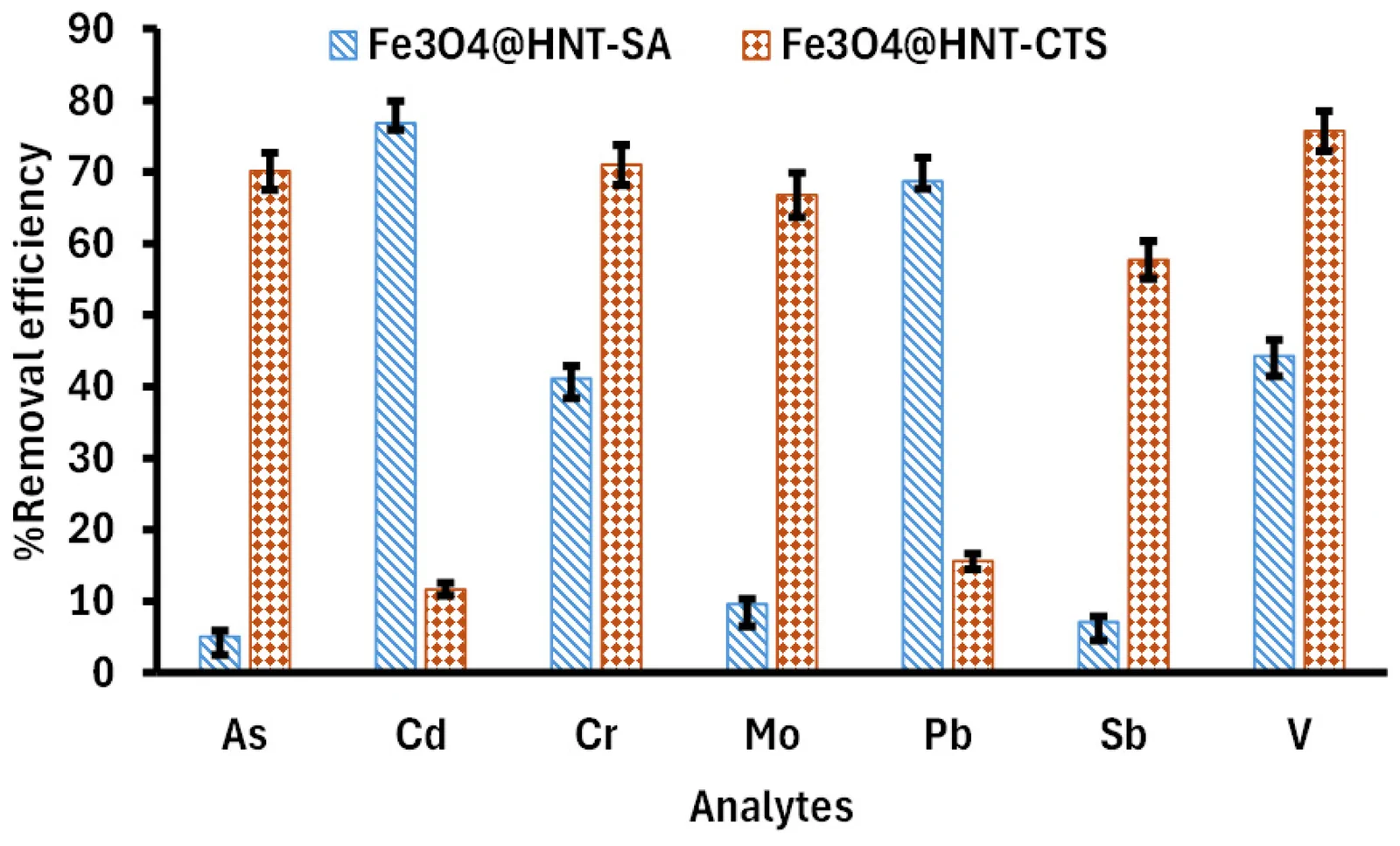
Selection of adsorbents for the adsorption of As, Cd, Cr, Mo, Pb, Sb, and V onto Fe_3_O_4_@HNT-SA and Fe_3_O_4_@HNT-CTS hydrogels. Experimental conditions: Sample volume: 30 mL; initial concentration: 1.0 mg/L; sample pH: 5.5; mass of adsorbent: 50 mg; contact time: 30 min.

The suitability of Fe_3_O_4_@HNT-SA and Fe_3_O_4_@HNT-CTS hydrogels was evaluated for the adsorptive removal of As, Cd, Cr, Mo, Pb, Sb, and V. As seen in Figure 7, the removal efficiency of Fe_3_O_4_@HNT-CTS performed better for the removal of As, Cr, Mo, Sb, and V compared to Fe_3_O_4_@HNT-SA. Consequently, Fe_3_O_4_@HNT-SA had higher adsorption efficiencies for Cd and Pb compared to Fe_3_O_4_@HNT-CTS. The results also show that the Fe_3_O_4_@HNT-SA has some affinity for Cr and V, but its adsorption performance does not surpass that of Fe_3_O_4_@HNT-CTS. The variation in the %RE of these Fe_3_O_4_@HNT-SA and Fe_3_O_4_@HNT-CTS hydrogels arises from their distinct surface properties. The pH of the solution also influenced their different performances. At the pH of 5.5, the speciation of the oxyanions is as follows: vanadate (H_2_VO_4_^−^) [36], molybdate (HMoO_4_^−^) [37], arsenous acid (H_3_AsO_3_) [38], and chromium (Cr^3+^) [39], Sb(OH)_3_. The functional groups (nitrogen, carboxyl and hydroxyl) on the Fe_3_O_4_@HNT-CTS surface at a pH of 5.5 can remove positively charged metals such as Cr^3+^ through complexation or chelation mechanisms. The species of V and Mo are negatively charged at pH 5.5 and, therefore, most likely to be adsorbed onto the positively charged surface of Fe_3_O_4_@HNT-CTS.

Meanwhile, the neutral species As and Sb interact with the protonated functional groups on the adsorbent through their oxygen lone pairs of electrons. The adsorption of Cd and Pb on Fe_3_O_4_@HNT-CTS was minimal due to the electrostatic repulsion between the positively charged absorbent and cations. The minimal adsorption observed might be because the surface area of Fe_3_O_4_@HNT-CTS was higher than that of Fe_3_O_4_@HNT-SA, resulting in higher surface activity. The negative surface of Fe_3_O_4_@HNT-SA repels (H_2_VO_4_^−^) and HMoO_4_^−^ and reduces their interaction with the material. In contrast, Cr demonstrated a higher removal percentage because it is positively charged around a pH of 5.5 and can interact with the deprotonated groups, hydroxyl and carboxyl groups. The two species (H_3_AsO_3_) and Sb(OH)_3_ are neutral species and lack the charge necessary for effective interaction with the deprotonated hydroxyl and carboxyl groups. The high concentration of hydroxyl and carboxyl groups can attract cations through electrostatic forces. This adsorbent was found to be more suitable for the adsorption of Cd and Pb, which benefited from the negative charge.

### 2.3. Optimisation of Batch Adsorption Experiments Using Central Composite Design

**Figure 8:**
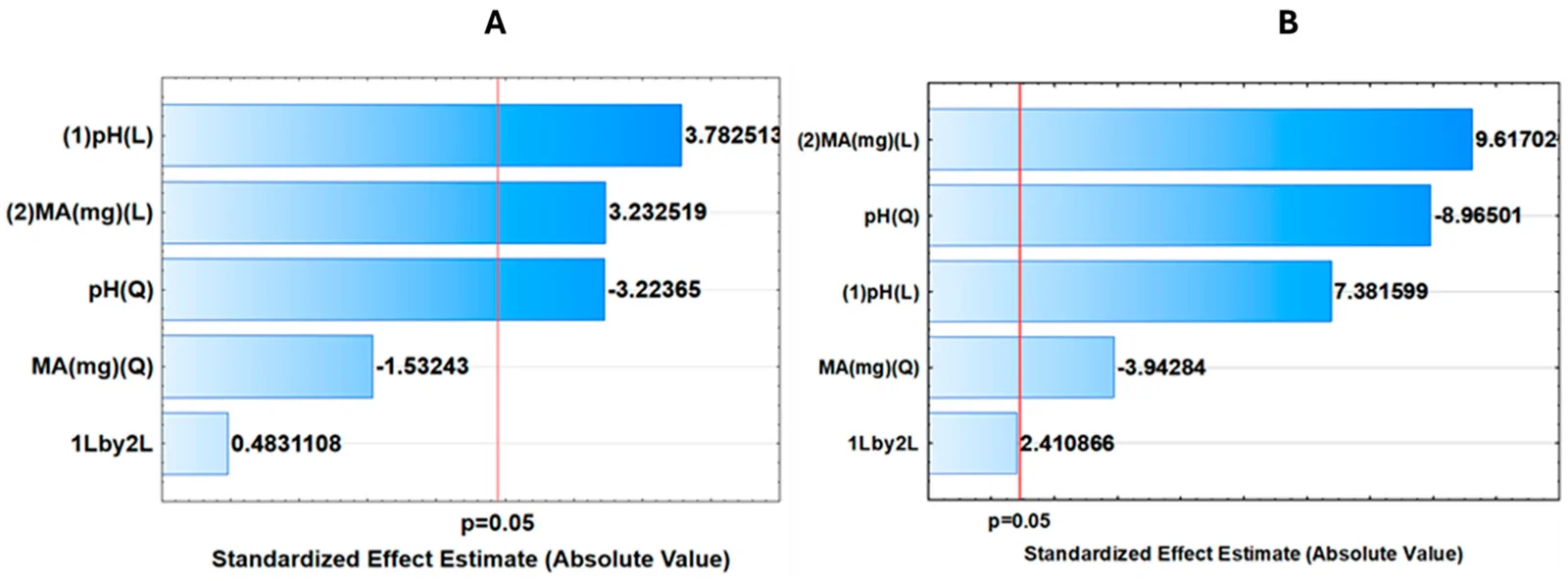
Pareto chart of (**A**) Fe_3_O_4_@HNT-SA (**B**) Fe_3_O_4_@HNT-CTS.

Central composite design (CCD) optimisation modelling was employed to identify the factors influencing the removal of Cd and Pb using Fe_3_O_4_@-SA-HNT, as well as the removal of As, Cr, Mo, Sb, and V using Fe_3_O_4_@CTS-HNT. The Analysis of variance (ANOVA) results are illustrated in the Pareto chart shown in Figure 8. This figure indicates that pH and MA were statistically significant for both adsorbents, as evidenced by their bars exceeding the vertical line. The pH is crucial due to its influence on the surface charge of the adsorbent and the chemical species of metals and metalloids present. MA is also essential since it determines the number of active sites available for reaction with the analytes.

**Figure 9:**
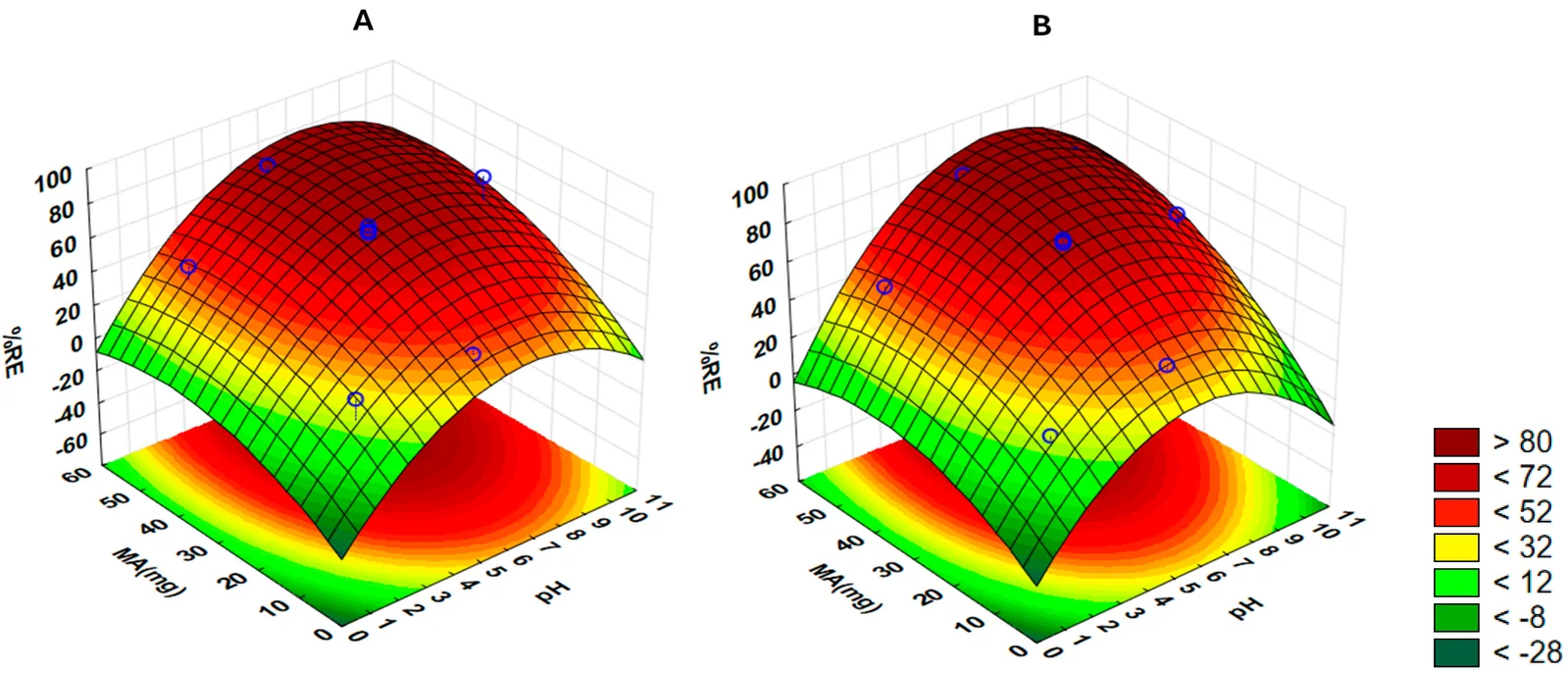
Response surfaces for combined effects of pH and MA for the removal of (**A**) Cd and Pb using Fe_3_O_4_@HNT-SA (**B**) As, Cr, Mo, Sb and V using Fe_3_O_4_@HNT-CTS.

Figure 9 presents the combined effect of sample pH and MA on Cd and Pb adsorption onto Fe_3_O_4_@HNT-SA adsorbent. Figure 9A illustrates that the adsorption of Cd and Pb onto the Fe_3_O_4_@HNT-SA surface increased with rising sample pH. The pH significantly influences the adsorption of Cd and Pb. At lower pH levels, the concentration of H^+^ is high, which leads to competition with the positively charged Cd and Pb ions, resulting in decreased removal efficiency. However, as the pH increases, %R also increases due to the higher availability of hydroxyl and carboxyl groups that facilitate the removal of Cd and Pb. Figure 9A also indicates that MA had no effect, but high MA increases the sites available for Cd and Pb adsorption [40].

Figure 9B presents the combined effect of sample pH and MA for the adsorption of As, Cr, Mo, Sb and V onto Fe_3_O_4_@HNT-CTS. The figure shows that when the pH is increased, the % R also increases. This is because the pH also determines the existence of As, Cr, Mo, Sb and V, and some exist as anionic species (H_2_VO_4_^−^, HMoO_4_^−^) at higher pHs, which are more easily adsorbed at acidic pH. The H^+^ decreases with increased pH, so there will be less competition between H^+^ and positively charged species (Cr^3+^). The -OH, -NH_2_ and Si-OH of Fe_3_O_4_@HNT-CTS deprotonate at higher pH, enabling electrostatic interaction between the ions and these functional groups. The adsorbent dosage did not affect the %R of As, Cr, Mo, Sb and V.

**Figure 10:**
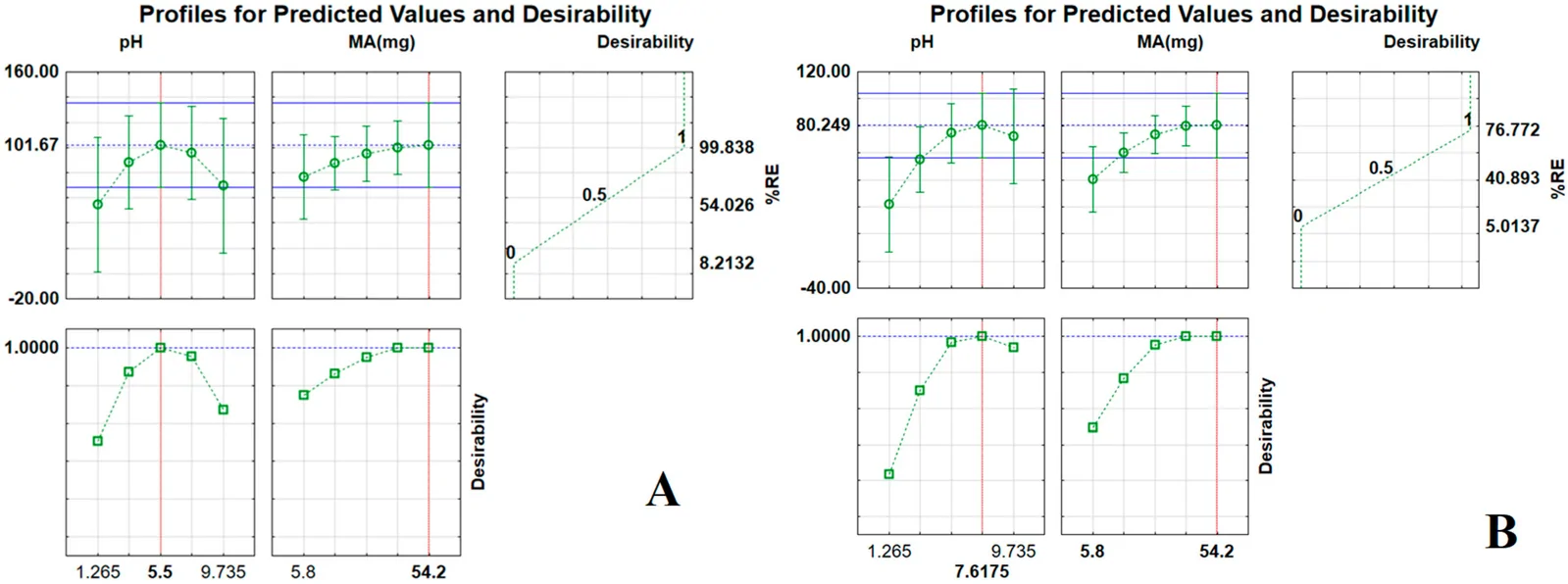
Predicted values and desirability function for removal of (**A**) As, Cr, Mo, Sb and V using Fe_3_O_4_@HNT-CTS (**B**) Cd and Pb using Fe_3_O_4_@HNT-SA.

Figure 10 illustrates the evaluation of the adsorption of Cd, Pb, V, Cr, Mo, As, and Sb ions using desirability functions. The desirability function for Fe_3_O_4_@HNT-SA, with a score of 1, had a maximum removal of 77%, and the lowest removal (5.0%) was scored 0. The intermediate was 0.5, with a removal percentage of 40.9%. The desirability function of Fe_3_O_4_@HNT-CTS scored 1 with a maximum removal of 83%, and the lowest removal (23%) was scored 0, with the intermediate being 0.5, which had a removal percentage of 52.7. A desirability score of 1 was targeted to achieve the best removal conditions. In comparison, the desirability function of Fe_3_O_4_@HNT-SA achieved a maximum removal efficiency (%RE) of 76.8 at a score of 1. At the intermediate score of 0.5, the %RE was 40.89, while the lowest score of 0 corresponded to a %RE of 5.01. Therefore, a desirability score of 1 was selected to obtain the maximum %RE. The Experimental validation with six replicates was performed using optimum conditions (pH = 5.5, MA = 40 mg and ET = 30 min) for the removal of As, Cr, Mo, Sb and V using Fe_3_O_4_@HNT-CTS yielded 70.2–93.3%RE While the optimum conditions (pH = 6.5–7.5, MA = 42mg and ET = 30 min) for the removal of Cd and Pb yielded an average %RE of 90.5–99.7%, demonstrating the surface methodology’s accuracy in determining optimal conditions.

### 2.4. Adsorption Studies

#### 2.4.1. Adsorption Isotherms Studies

**Figure 11:**
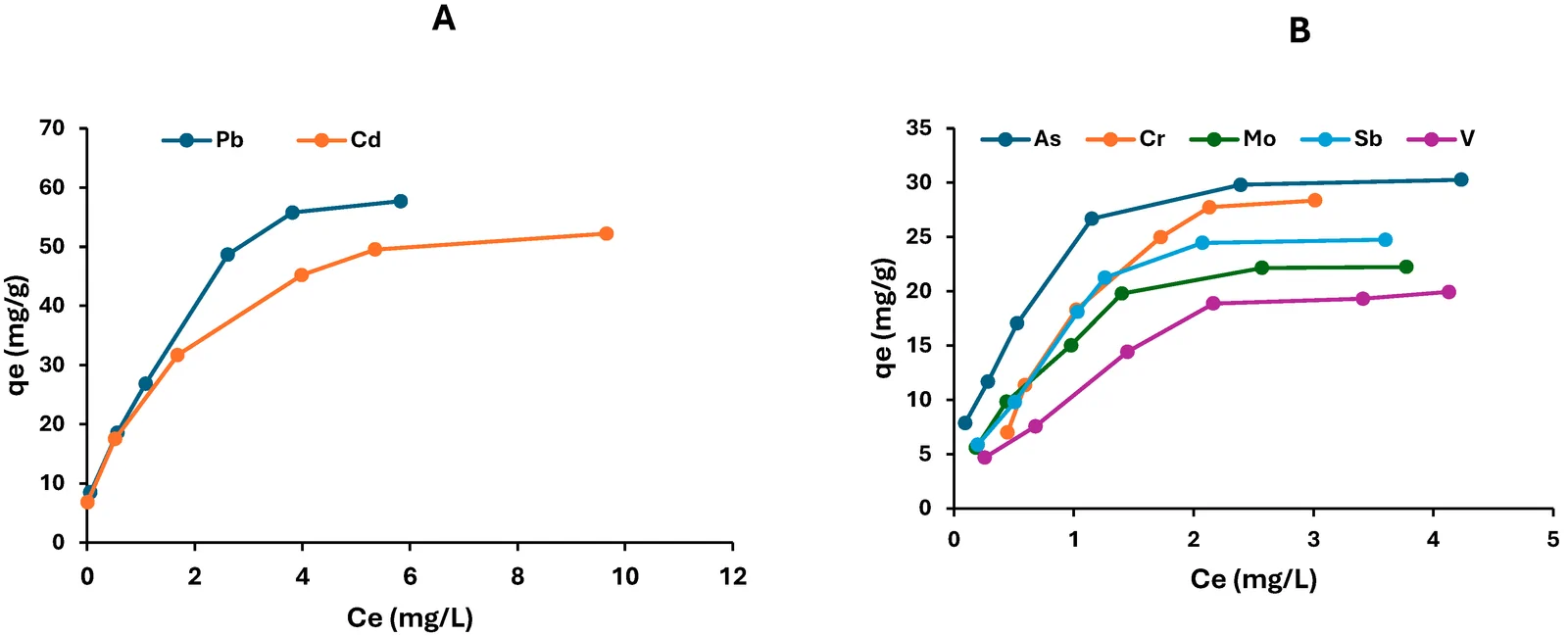
Equilibrium data for adsorption of (**A**) Cd, Pb, experimental conditions pH = 7.5, MA = 42mg and ET = 30 min, (**B**) As, Cr, Mo, Sb and V with experimental conditions: pH = 5.5, MA = 40mg and ET = 30 min.

The equilibrium isotherm studies investigated the interaction between the Fe_3_O_4_@HNT-SA and Fe_3_O_4_@HNT-CTS adsorbents and their respective adsorbates. The relationship between Cd, Pb and the Fe_3_O_4_@HNT-SA is illustrated in Figure 11A. In contrast, the relationship between As, Cr, Mo, Sb and V with the Fe_3_O_4_@HNT-CTS is shown in Figure 11B. As depicted in both figures, the adsorption capacity of the analytes increases with higher initial metal ion concentrations. The presence of active sites on the surface of the Fe_3_O_4_@HNT and Fe_3_O_4_@HNT-CTS increased the amount adsorbed (qe) at lower initial concentrations. However, the active sites reach equilibrium as the initial concentrations rise due to the saturation of the binding sites. The data were analysed using Langmuir and the Freundlich isotherm models, and their parameters are summarised in Table 2.

The regression correlation coefficients and isotherm model parameters were calculated using linearised Langmuir and Freundlich expressions from Figures S3 and S4, and the parameters are shown in Table 2.

Langmuir mode1:

(1) $$\frac{C_{e}}{q_{e}}=\frac{1}{K_{L}q_{max}}+\frac{C_{e}}{q_{max}}$$

where *q_max_* is the maximum adsorption capacity (mg/g), and *R_L_* is the Langmuir constant (L/mg).

(2) $$R_{L}=\frac{1}{1+K_{L}c_{o}}$$

Freundlich model:

(3) $$lnq_{e}=lnk_{f}+\frac{1}{n}lnc_{e}$$

*K_f_* (mg/g) is the Freundlich constant, and 1/*n* is the heterogeneity factor.

The R^2^ value was used to determine which model provided a better fit. The R^2^ values for As, Cr, Sb, Mo, and V were high for the Langmuir model, ranging from 0.96 to 0.99. This suggests that the adsorption processes occurred on homogeneous sites of Fe_3_O_4_@HNT-CTS [3]. In contrast, Cr showed high R^2^ (0.94) for the Freundlich model instead, indicating multilayered adsorption [41]. The adsorption of Cd by Fe_3_O_4_@HNT-SA exhibited a high R^2^ (0.98) for the Langmuir model, indicating its adsorption occurred on a uniform surface. in contrast, Pb displayed a similarly high R^2^ value (0.98) for the Freundlich model, suggesting that the adsorption happened on a heterogeneous surface [41].

The maximum capacities of As, Cd, Mo, Pb, Sb and V were closer to the experimental e, suggesting that the Langmuir model explained the adsorption data better. The parameter R_L_ was used to evaluate the nature of the adsorption process, categorising it as unfavourable (*R_L_* > 1), linear (*R_L_* = 1), favourable (0 < *RL* < 1), or irreversible (*R_L_* = 0) [42]. All analytes measured had RL values that were less than one but greater than zero, indicating that their adsorption was favourable. The Freundlich constant (*KF*) reflects the degree of affinity between the analytes and the adsorbents, with higher *KF* values signifying a stronger affinity [43]. The order of affinity towards Fe_3_O_4_@HNT-CTS was found to be: Cr > Sb > As > V > Mo. In contrast, Pb exhibited a greater affinity for Fe_3_O_4_@HNT-SA compared to Cd.

#### 2.4.2. Adsorption Kinetics Studies

**Figure 12:**
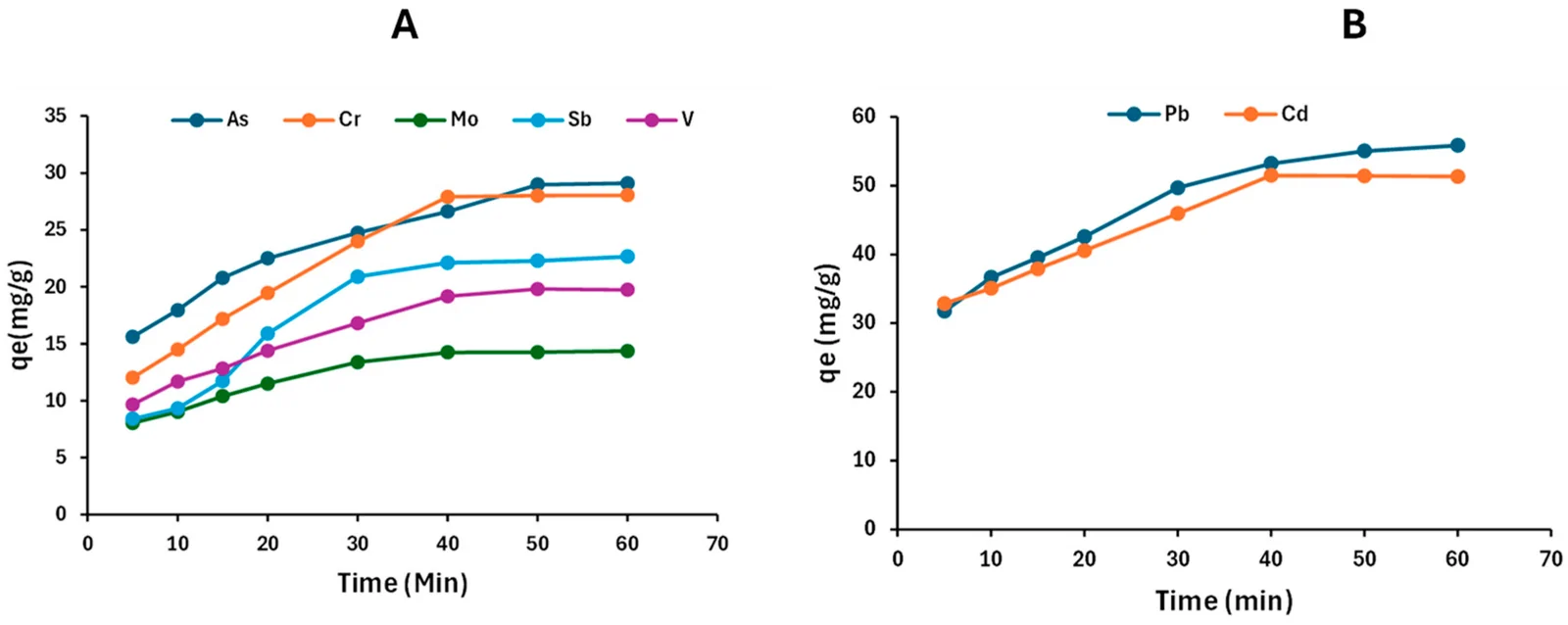
Equilibrium data for adsorption of (**A**) As, Cr, Mo, Sb and V with experimental conditions: pH = 5.5, MA = 40 mg and initial concentration = 9 mg/L and (**B**) Cd, Pb, experimental conditions pH = 7.5, MA = 42 mg and initial concentration = 17 mg/L.

Adsorption kinetics is a key measurement that evaluates the adsorbent’s effectiveness in the adsorption rate. This rate is significantly influenced by the diffusion process and the contact time of the adsorbate at the solid-solution interface [44]. Figure 12 presents the relationship between adsorption capacity and contact time. Figure 12A shows that between 0 and 40 min, Fe_3_O_4_@HNT-CTS provided sufficient active sites for interactions, facilitating rapid adsorption of As, Cr, Mo, Sb, and V. Then, after 40 min, the adsorptive sites were saturated, and equilibrium was reached. In Figure 12B, it is observed that Cd and Pb reached equilibrium within 40 min as well.

The pseudo-first-order and pseudo-second-order models were utilised, and the findings are displayed in Table 3. The parameters were calculated from Figures S6 and S5.

Pseudo-first order:

(4) $$\ln\left(q_{e}-q_{t}\right)=\ln(K_{1}q_{e})-K_{1}t$$

Pseudo-second order:

(5) $$\frac{t}{q_{t}}=\frac{1}{K_{2}q_{e^{2}}}+\frac{1}{q_{e}}$$

According to Table 3, all the analytes had the highest correlation coefficient (R^2^) for the pseudo-second-order model, and As fitted the pseudo-first-order model better. The pseudo-second-order model calculated qe values for As, Cd, Cr, Mo, Pb, Sb, and V were closer to the experimental qe values.

Also, similarly, As and Pb adsorption can be described by the pseudo-second-order model, as the R^2^ values are greater than 0.99.

#### 2.4.3. Thermodynamics

The effect of temperature on the removal efficiency of Cd and Pb using Fe_3_O_4_@HNT-SA, and As, Cr, Mo, Sb, and V using Fe_3_O_4_@HNT-CTS, was investigated. The thermodynamic parameters were evaluated under optimum adsorption conditions, considering both equilibrium concentration and equilibrium time. The temperature range studied was 290–313 K. Thermodynamic parameters, including Gibbs free energy (ΔG°), enthalpy (ΔH°), and entropy (ΔS°), were calculated using Equations (6) and (7).

(6) ∆G°=−RTlnK

(7) lnK=∆S°/R−DH°/RT

(8) $$K=Q_{e}/C_{e}$$

The universal gas constant is (8.314 J mol^−1^ K^−1^), while T refers to the absolute temperature in kelvins (K), and Kc denotes the equilibrium constant associated with the adsorption process.

The enthalpy change (ΔH°) for Pb, As, Cr, Mo, and V with their respective adsorbents, as shown in Table 4, was positive, indicating that the adsorption process was endothermic. Therefore, increasing the temperature enhanced the adsorption of these analytes. In contrast, Cd and possibly V adsorption was exothermic (ΔH° < 0), meaning lower temperatures favoured their adsorption. The entropy change (ΔS°) for Pb, As, Cr, Mo, and V was positive, suggesting increased randomness at the solid–solution interface and their spontaneous adsorption processes. In contrast, Cd and V exhibited negative ΔS° values, implying a decrease in randomness and less favourable adsorption behaviour, and the reaction needed energy for initiation [44]. The values of ΔG° for all analytes were less than 20 kJ/mol, showing that the analytes were physically adsorbed [45].

### 2.5. Interference Studies

The interference studies were conducted on Cd and Pb using Fe_3_O_4_@HNT-SA, and on As, Cr, Mo, Sb, and V using Fe_3_O_4_@HNT-CTS, using optimum conditions. The goal was to evaluate whether the coexisting species commonly found in water could influence the adsorption of the target analytes. To carry out the experiments, 20 mg/L concentrations of each interfering ion were spiked or added to the standard solutions of the target ions mentioned above. In Table S1, it is shown that the other ions (Mg, Na, Cr) have no noticeable impact (above 5%) on the removal of Cd and Pb. However, when zinc was added, there was a significant decrease in the percentage removal of these metals, dropping from 95.89% to 84.97%. This decline is attributed to Zn, which has a comparable ionic radius and also has a good affinity for the OH group on the surface of the Fe_3_O_4_@HNT-SA, which, as a result, competes with the target analytes for the active sites [46]. The interference study for As, Cr, Mo, Sb, and V onto Fe_3_O_4_@HNT-CTS was carried out with interference ions Zn, Mg, Na, Pb, Cd, and Fe using the optimum conditions. The interference ions of concentration of 20 ppm were each added to standard solutions containing the target ions. The increase in the concentrations of these interfering ions had a slight impact on the percentage removal of the target analytes. It was found that the investigated interferences did not have a significant effect, as the difference was less than 5% as indicated by Table S2. These finding was further supported by the analysis of real river water samples, where the removal percentage was not affected by the presence of possible interference studied above.

### 2.6. Reusability Studies

**Figure 13:**
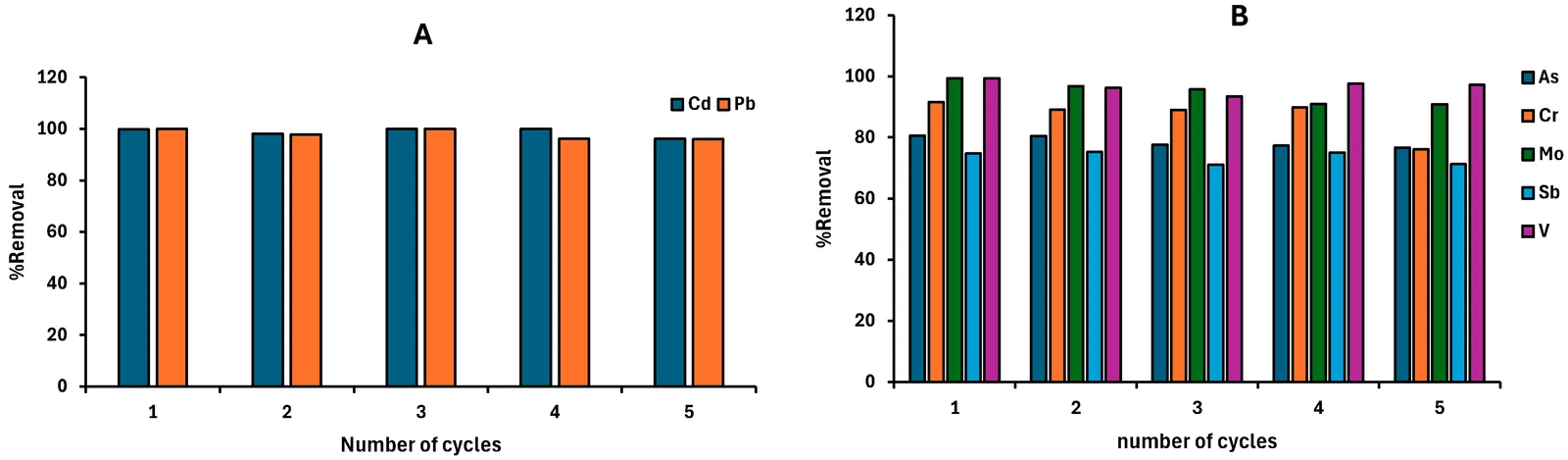
Recyclability study of Fe_3_O_4_@HNT-SA for (**A**) Cd and Pb removal and (**B**) Fe_3_O_4_@HNT-CTS for As, Cr, Mo, Sb, and V removal.

The efficacy of the two adsorbents was evaluated for reuse to determine if there was a significant change in the removal percentage after multiple uses. As shown in Figure 13A, Fe_3_O_4_@HNT-SA was reused five times for the removal process without a significant change in effectiveness. In contrast, Figure 13B indicates that Fe_3_O_4_@HNT-CTS had a decrease in %removal for As, Mo and V, with a difference of less than 5%. For chromium, the removal efficiency declined from 91.59% in the first cycle to 76.1% in the last cycle, showing a significant decrease. Similarly, antimony also experienced a noticeable decline, dropping from 74.65% to 65.26% by the fifth cycle. This decline may be attributed to the adsorbent containing a small portion of these two analytes after each adsorption/desorption cycle, which likely led to the blockage of active sites after each successive cycle. Therefore, it can be concluded that both Fe_3_O_4_@HNT-SA and Fe_3_O_4_@HNT-CTS can be effectively used for up to five cycles while achieving acceptable removal percentages. This finding also suggests that the materials are cost-effective.

### 2.7. Application in Real Water Samples

The applicability of the prepared adsorbents Fe_3_O_4_@HNT-CTS and Fe_3_O_4_@HNT-SA was assessed by analysing five samples of water from the river spiked with known concentrations of the target analytes. The results showed that the removal efficiencies of Fe_3_O_4_@HNT-CTS and Fe_3_O_4_@HNT-SA effectively removed the target analytes. The concentration in the final solution was not detected, suggesting that the Fe_3_O_4_@HNT-CTS and Fe_3_O_4_@HNT-SA are suitable adsorbents for treating contaminated surface water. The concentrations were reduced to levels that are below the World Health Organisation (WHO) permissible limits for various contaminants in drinking water [47,48,49,50,51,52].

### 2.8. Comparison Studies

Table 5 summarises a comparison of Fe_3_O_4_@HNT-CTS and Fe_3_O_4_@HNT-SA with other adsorbents for the removal of metallic elements. As shown in the table, these two adsorbents were evaluated against various other adsorbents. The Fe_3_O_4_@HNT-SA exhibited a higher adsorption capacity compared to polyethyleneimine (PEI) cryogels, which had capacities ranging from 19.88 to 24.39 mg/g [53], and nano-sized SiO_2_, with capacities of 42.2 mg/g and 34.2 mg/g for the adsorptive removal of Cd and Pb [54]. Additionally, modified activated carbon for V achieved a capacity of 19.45 mg/g [55], which is smaller than the adsorption capacity of 24.7 mg/g achieved with Fe_3_O_4_@HNT-CTS. However, adsorbents such as a manganese-residues/serpentine-based composite reached 98.05 mg/g for Cd and 565.81 mg/g for Pb were significantly higher [56]. Metal–organic frameworks (MOFs) also attained a very high adsorption capacity of 188.12 mg/g for Cr and 349.09 mg/g for Pb [57]. Although the synthesised adsorbents have lower adsorption capacities compared to most of those listed in the table, their eco-friendliness and cost-effectiveness remain strong selling points, especially when considering that they are derived from natural sources.

## 3. Experimental

### 3.1. Materials and Chemicals

Halloysite nano clay (H_2_O_9_Si_2_·2H_2_O) (1.26–1.34 mL/g), iron(II) sulphate (ACS reagent ≥ 99%, FeSO_4_·7H_2_O) and iron(III) chloride hexahydrate (FeCl_3_·6H_2_O), ammonium hydroxide (~25%, NH_4_OH), and ultrapure nitric acid (65–70% *w*/*w*, HNO_3_, ultra-trace) were purchased from Merck (Johannesburg, South Africa). Sodium hydroxide (NaOH), ultrapure hydrochloric acid (37% *w*/*w*, HCl. 1000 mg L^−1^ standards of Cd, Cr, Pb, and Sb were bought from Sigma (South Africa, Gauteng). Chitosan (C_12_H_24_N_2_O_9_) and alginate were purchased from Sigma. The Mo, V, As and Sb standards were bought from Spectroscan Teknolab, Norway.

### 3.2. Instrumentation

Functional groups of Fe_3_O_4_@HNT-SA and Fe_3_O_4_@HNT-CTS were characterised using a PerkinElmer Fourier transform infrared spectroscopy (FTIR) system (KBr) with scans ranging from 400 to 4000 cm^−1^, located in Waltham, MA, USA. The scanning electron microscopy (SEM) coupled with energy dispersive spectrometry (EDS) SEM, TESCAN VEGA 3 XMU, LMH instrument from Bruno, Czech Republic, determined the morphology and elemental composition of the adsorbents. Transmission electron microscopy (TEM) was employed to delve deeper into the morphology of Fe_3_O_4_@HNT-SA and Fe_3_O_4_@HNT-CTS, using a TEM JEM-2100 obtained from JOEL Ltd, Tokyo, Japan. A SciTech ultrasonic bath system, which operates at 50 Hz and 150 W, was provided by Labotec in Midrand, South Africa, for the adsorption processes for As, Cd, Cr, Mo, Pb, Sb and V. The analyte residuals were analysed by inductively coupled plasma-optical emission spectrometer (ICP-OES) (model iCAP 6500 Duo from Thermo Scientific, Birmingham, UK). The crystal structures of Fe_3_O_4_@HNT-SA and Fe_3_O_4_@HNT-CTS were explored through X-ray powder diffraction (XRD) techniques using instruments from PANalytical (Almelo, The Netherlands). Surface area and pore size measurements were conducted employing the Brunauer–Emmett–Teller (BET) method with an ASAP2020 V3.00H unit from Micromeritics Instrument Corporation in Norcross, GA, USA. The zetasizer Instrument from Malvern (UK) was used to measure the zeta potential of Fe_3_O_4_@HNT-SA and Fe_3_O_4_@HNT-CTS. The experimental data were analysed with STATISTICA software version 14.

### 3.3. Synthesis of Magnetic Halloysite Nanotube (HNT@Fe_3_O_4_)

The magnetic halloysite nanotube (HNT@Fe_3_O_4_) was synthesised by modification of the previously reported method [61]. Approximately 4.8 g of FeCl_3_·6H_2_O and 2.4 g of FeSO_4_·7H_2_O were dissolved in 50 mL of water. Then, on the side, HNT powder (1 g) was dispersed in 100 mL of water by sonication for 30 min. The two mixtures were combined and stirred, then purged with nitrogen gas for 10 min at 60 °C. Then (~25%) of the ammonium hydroxide was added dropwise into the mixture, and the black precipitate (HNT@Fe_3_O_4_) formed. The mixture was left to age for 4 h at 70 °C. An external magnet was used to separate the black residue of HNT@Fe_3_O_4_, which was then washed several times with water. The prepared HNT@Fe_3_O_4_ was subsequently dried overnight in an oven at 60 °C.

### 3.4. Synthesis of HNT@Fe_3_O_4_-CTS

The HNT@Fe_3_O_4_-CTS was synthesised as follows: 2 g of HNT@Fe_3_O_4_ was added to 100 mL of ultrapure water and stirred at 500 rpm. In a separate beaker, 2.0 g of CTS was dissolved in 100 mL of 1% acetic acid solution. Once the CTS was fully dissolved, it was added drop by drop to the HNT@Fe_3_O_4_ solution and stirred for 24 h to obtain the HNT@Fe_3_O_4_-CTS composite. Then, acetic acid was removed by washing HNT@Fe_3_O_4_-CTS with water several times [62].

### 3.5. Preparation of Fe_3_O_4_@HNT-SA

The Fe_3_O_4_@HNT-SA nanocomposite was prepared using the co-precipitation method. First, 1.0 g of SA polymer was dissolved in 150 mL of ultrapure water. In a separate beaker, 1.0 g of HNT was dispersed in 50 mL of water and mixed with the SA solution. Next, the solution was mixed with 50 mL of FeCl_3_·6H_2_O (4.8 g) and FeSO_4_·7H_2_O (2.4 g) (forming a brown gel). The mixture was then heated at 70 °C and protected with nitrogen gas for 30 min. Then, 50 mL (~28%) of ammonium hydroxide was added, forming a black precipitate (Fe_3_O_4_@HNT-SA). The mixture was left to age for 2 h at 70 °C. The particles were then collected with an external magnet, washed multiple times with water, and dried overnight at 60 °C.

### 3.6. Batch Adsorption Experiments

The adsorption of Cd and Pb using Fe_3_O_4_@HNT-SA and As, Cr, Mo, Sb, and V by Fe_3_O_4_@HNT-CTS was conducted using batch experiments. The method was carried out by adding 30 mL of a solution (pH of 1.26–9.74, adjusted with 0.5 mol/L NaOH/HCl) containing mixtures of As, Cr, Mo, Sb, and V or Cd and Pb at 1.0 mg/L of Cd and Pb into 50 mL centrifuge tubes containing 5.78–54.2 mg of either Fe_3_O_4_@HNT-CTS or Fe_3_O_4_@HNT-SA hydrogel. The mixture was sonicated for 30 min, and the adsorbent was separated by magnetic decantation. The aqueous solution was filtered using 0.22 µm PVDF syringe filter membranes, and the residual elemental concentrations were analysed with ICP-OES. Each experiment was performed three times.

The suitability of Fe_3_O_4_@HNT-CTS and Fe_3_O_4_@HNT-SA hydrogels for the simultaneous removal of the analytes of interest was evaluated using the method mentioned above. The experimental conditions were fixed as follows: 30 mL, 1.0 mg/L, 5.5, 50 mg, and 30 min for sample volume, initial concentration, sample pH, mass of adsorbent, and contact time, respectively.

Equation (1) was used to calculate the percentage removal efficiency, where C_i_ and C_e_ were elemental concentrations before and after adsorption.

(9) $$\%R=\frac{C_{0}-C_{e}}{C_{e}}\times 100$$

The central composite design (CCD) was used to investigate the most significant factors for the removal of As, Cd, Cr, Mo, Pb, Sb and V using respective hydrogel material. The independent factors (mass of adsorbent (MA) and sample pH) were examined at five levels denoted as −α, −1, 0, +1, and +α, representing the chosen values from the lowest to the highest. The factor levels and their experimental domains are presented in Table 6.

### 3.7. Adsorption Kinetics and Isotherm Studies

The kinetics studies were investigated using two commonly used linear expressions, such as pseudo-first-order and pseudo-second-order, to explain the adsorption of As, Cd, Cr, Mo, Pb, Sb and V onto Fe_3_O_4_@HNT-CTS and Fe_3_O_4_@HNT-SA hydrogels. The batch adsorption experiments were conducted at optimum conditions to investigate the effect of contact time on the adsorption capacities of Fe_3_O_4_@HNT-CTS and Fe_3_O_4_@HNT-SA hydrogels for the target analytes. The adsorption process was performed for 5–60 min using the optimised method. The adsorption of As, Cr, Mo, Sb and V onto Fe_3_O_4_@HNT-CTS hydrogel was carried out using sample pH, mass of adsorbent, and sample volume of 5.5, 40 mg and 300 mL, respectively. For the adsorption of Cd and Pb onto Fe_3_O_4_@HNT-SA hydrogel, a sample pH ranging from 6.5 to 7.0 was used.

Linear equations of Langmuir and the Freundlich isotherms were used to describe the equilibrium data. Therefore, the effect of elemental concentrations on the adsorption capacities of Fe_3_O_4_@HNT-CTS and Fe_3_O_4_@HNT-SA hydrogels was investigated in the range of 1–9 mg/L (As, Cr, Mo, Sb and V) and 1–25 mg/L (Cd and Pb). It is important to note that the elemental concentrations in the aqueous solutions were determined by ICP-OES. The adsorption data for isotherms and kinetics were calculated using Equations (10) and (11).

(10) $$q_{e}=\frac{C_{o}-C_{e}}{m}\times V$$

(11) $$q_{t}=\frac{C_{o}-C_{t}}{m}\times V$$

where q_e_, q_t_, V, m, C_e_ and C_t_ were the equilibrium adsorption capacity (mg/g), equilibrium capacity (mg/g) at time t, sample volume (L), mass of adsorbent (g), elemental concentrations (mg/L) at equilibrium and time t, respectively.

### 3.8. Reusability

A total of 42 mg of Fe_3_O_4_@HNT-SA was added to the centrifuge. Following that, 30 mL of a sample solution containing Cd and Pb was also added to the centrifuge. The mixture was sonicated for 30 min. After sonication, the supernatant was separated, filtered, and analysed using ICP-OES. The recovered adsorbent was then washed with 2M HNO_3_ to eliminate any adsorbed analytes, followed by washing with NaOH and then with water. This process allowed the adsorbent to be reused up to five times. In a separate centrifuge tube, 40 mg of Fe_3_O_4_@HNT-CTS was added, followed by the addition of 30 mL of a sample solution containing analytes (As, Cr, Mo, Sb, and V). The mixture was sonicated for 30 min, after which the supernatant was filtered and analysed using ICP-OES. The adsorbed analytes were then removed with 10 mL of a 1:1 HCl/HNO_3_ solution and subsequently neutralised with 1 mL of 1 M NaOH. Finally, the mixture was washed several times with water.

### 3.9. Application to Real Water Samples

The applicability of the hydrogels was evaluated as an adsorbent for the extraction of specific analytes from river water samples taken from various villages in the Eastern Cape Province of South Africa. Optimised adsorption processes were employed for this purpose. To remove As, Cr, Mo, Sb, and V using Fe_3_O_4_@HNT-CTS adsorbent, the pH of the river samples was adjusted to 5.5. In contrast, the water samples were not adjusted because their pH was between 6.5 and 7.5 during the removal of Cd and Pb. The samples were then separated using an external magnet, filtered, and analysed using ICP-OES.

## 4. Conclusions

This study successfully utilised naturally occurring materials to create low-cost and environmentally friendly adsorbents with different functionalities to remove two sets of metallic pollutants: oxyanions (As, Cr, Mo, Sb and V) and metals (Cd and Pb). The Fe_3_O_4_@HNT-biopolymer demonstrated versatility in removing toxic ions. The Fe_3_O_4_@HNT-SA demonstrated its maximum removal efficiency at a pH of 7.5, while Fe_3_O_4_@HNT-CTS showed optimal performance at pH 5.5. The adsorption of this study was strongly dependent on pH, showing the interaction between metal ion speciation and protonation/deprotonation of the surface groups of the adsorbents. The adsorption of most analytes was governed by Langmuir models, suggesting that the reaction happens on a homogeneous surface of the adsorbent.

Additionally, Cr and Pb fit well within the Freundlich model. The two adsorbents also demonstrated in the interference studies that they can remove the target analyte in the presence of other or competing ions, and their application in the river water highlighted the applicability of this method. Integrating Halloysite nanoclay with biopolymers offers a sustainable and environmentally friendly approach to remediating toxic metals from the environment.

## Figures and Tables

**Table 1:** Textual properties of SA, HNT, CTS, Fe_3_O_4_, Fe_3_O_4_@HNT-SA and Fe_3_O_4_@HNT-CTS.

Adsorbent	Surface Area (m^2^/g)	Pore Volume (cm^3^/g)	Average Pore Size (nm)
CTS	1.60	0.00499	8.57
SA	3.82	0.00231	7.46
HNT	60.4	0.345	13.1
Fe_3_O_4_	86.0	0.302	12.5
Fe_3_O_4_@HNT-CTS	58.4	0.179	9.85
Fe_3_O_4_@HNT-SA	40.7	0.144	11.2

**Table 2:** Linearised isotherm model parameters for the adsorption of As, Cr, Mo, Sb and V onto Fe_3_O_4_@HNT-CTS and the adsorption of Cd and Pb onto Fe_3_O_4_@HNT-SA.

Models	Parameters	As	V	Cr	Mo	Sb	Cd	Pb
	Fe_3_O_4_@HNT-CTS Hydrogel						Fe_3_O_4_@HNT-SA Hydrogel	
	q_e_ (expt) (mg/g)	30.3	19.9	28.4	22.2	24.7	52.2	57.7
Langmuir	q_max_ (mg/g)	33.60	27.2	69.9	27.0	31.7	56.2	69.4
	K_L_(L/mg)	2.48	0.74	0.246	1.46	1.19	1.22	59.9
	R_L_	0.0464	0.166	0.375	0.0922	0.109	0.0472	0.0735
	R^2^	0.9951	0.9693	0.6667	0.9920	0.9720	0.9861	0.9589
Freundlich	K_F_ (mg/g)	11.6	11.1	21.9	10.6	13.50	16.4	26.0
	N	0.331	0.429	0.385	0.378	0.367	0.304	0.301
	R^2^	0.9459	0.9574	0.9441	0.942	0.9254	0.9774	0.9788

**Table 3:** Linearised kinetic model parameters for the adsorption of Cd and Pb onto the Fe_3_O_4_@HNT-SA adsorbent and the adsorption of As, Cr, Mo, Sb and V using Fe_3_O_4_@HNT-CTS.

Models	Parameters	Cd	Pb	As	V	Cr	Mo	Sb
	q_e_, expt (mg/g)	52.2	57.7	30.3	19.9	28.4	22.2	24.7
Pseudo-first-order	*k*_1_ (1/min)	0.0706	0.0570	0.0302	0.0834	0.0843	0.0115	0.0429
	*q_e,calc_.*	34.14	36.34	20.2	21.9	33.3	14.0	20.3
	R^2^	0.8799	0.9935	0.9709	0.9310	0.9254	0.8873	0.9259
Pseudo-second-order	k_2_ (g/mg∙min)	0.00298	0.0220	0.0000575	0.00426	0.00219	0.00881	0.00243
	q_e_ (mg/g)	57.4	62.5	32.68	23.26	34.84	16.2	26.2
	R^2^	0.9937	0.9953	0.9946	0.9908	0.9857	0.9959	0.9598

**Table 4:** Thermodynamic parameters of adsorption of Cd and Pb onto the Fe_3_O_4_@HNT-SA adsorbent and the adsorption of As, Cr, Mo, Sb and V using Fe_3_O_4_@HNT-CTS.

Analytes	ΔG° (kj, mol^−1^)			ΔH° (kj, mol^−1^)	ΔS° (j, mol^−1^k^−1^)
	298 K	308 K	313 K		
Fe_3_O_4_@HNT-Cd	−4.30	−1.95	−0.768	−74.6	−236
Fe_3_O_4_@HNT-SA-Pb	−5.32	−6.36	−6.88	25.7	104
Fe_3_O_4_@HNT-CTS-As	−4.46	−4.85	−5.05	7.29	39.4
Fe_3_O_4_@HNT-CTS-Cr	−1.78	−2.18	−2.39	133	451
Fe_3_O_4_@HNT-CTS-Mo	−2.85	−4.51	−5.44	48.6	173
Fe_3_O_4_@HNT-CTS-Sb	−4.62	−2.52	−1.48	−67.1	−210
Fe_3_O_4_@HNT-CTS-V	−3.43	−5.04	−5.58	44.6	161

**Table 5:** Comparison of Fe_3_O_4_@HNT-CTS and Fe_3_O_4_@HNT-SA with other adsorbents for the removal of metals/metalloids.

Adsorbent	Analyte	Maximum Uptake (mg/g)	References
Nano-sized silicon dioxide	Cd and Pb	42.2 and 34.2	[54]
Polyethyleneimine (PEI) cryogels	Cd, Co, Cr, Ni, Pb, and Zn	19.88–24.39	[53]
manganese-residues-and-serpentine-based composite	Cd and Pb	98.05 and 565.81	[56]
Polyaniline-TiO_2_ hydrate:	Cr and Sb	394 and 48.5	[58]
Bacteria–mediated kaolin@Fe–Mn binary hydroxides	Sb(III), Sb(V), As(III) and As(V)	177.19, 56.26, 62.92 and 42.18	[59]
metal–organic frameworks	Cr and Pb	188.12 and 349.09	[57]
	Sb	30.26, 86.35	[60]
modified activated carbon	V	19.45	[55,58]
Fe_3_O_4_@HNT-CTS	As, Cr, Mo, Sb, and V	30.3, 19.9, 28.4, 22.2, 24.7	This study
Fe_3_O_4_@HNT-SA	Cd and Pb	52.2 and 57.7	This study

**Table 6:** Factors and levels employed in central composite design for the removal of As, Cd, Cr, Mo, Pb, Sb and V.

Factors	−α	−1	0	+	+α
Mass adsorbent (MA, mg)	5.78	10	30	50	54.2
Sample pH	1.26	2.0	5.5	9.0	9.74

## Data Availability

The raw data supporting the conclusions of this article will be made available by the authors on request.

## Supplementary Materials

The following supporting information can be downloaded at: https://www.mdpi.com/article/10.3390/molecules30183777/s1, Figure S1: TEM images of (A) HNT and (B) Fe_3_O_4_. Figure S2: SEM images of (A) HNT (B) Fe_3_O_4_. Figure S3: (A) Langmuir isotherm plot for the adsorption of Cd and Pb using Fe_3_O_4_@HNT-SA, (B) Freundlich isotherm plot for Cd and Pb using Fe_3_O_4_@HNT-SA. Figure S4: (A) Langmuir isotherm plot for the adsorption of As, Cr, Mo, Sb and V using Fe_3_O_4_@HNT-CTS (B) Freundlich isotherm plot for As, Cr, Mo, Sb and V using Fe_3_O_4_@HNT-CTS. Figure S5: (A) Pseudo first order kinetics and (B) pseudo second order kinetics of Cd and Pb using Fe_3_O_4_@HNTSA adsorbent. Figure S6: (A) Pseudo first order kinetics and (B) pseudo second order kinetics of As, Cr, Mo, Sb and V using Fe_3_O_4_@HNT-CTS. Table S1. The effect of interfering studies on the adsorption of Cd and Pb using Fe_3_O_4_@HNT-SA. Table S2. The effect of interfering studies on the adsorption of As, Cr, Mo, Sb and V onto Fe_3_O_4_@HNT-CTS.

## Abbreviations

CCD	Central composite design
CTS	Chitosan
Fe_3_O_4_@HNT-CTS	Magnetic halloysite nanoclay chitosan
HNT	Halloysite nanotubes
ICP-OES	Inductively coupled plasma optical emission spectroscopy
Fe_3_O_4_@HNT-SA	Magnetic halloysite nanoclay alginate
NIP	Non-imprinted polymer
SA	Alginate
