# Supplementary material for: Removal of Oxyanions and Trace Metals from River Water Samples Using Magnetic Biopolymer/Halloysite Nanocomposites
Source: Molecules. 2025 Sep 17;30(18):3777. doi: 10.3390/molecules30183777 (PMC12472565; doi:10.3390/molecules30183777)
Supplement: Supplementary file 1 [file molecules-30-03777-s001.zip › molecules-3726180-supplementary.pdf]

Supplementary Materials

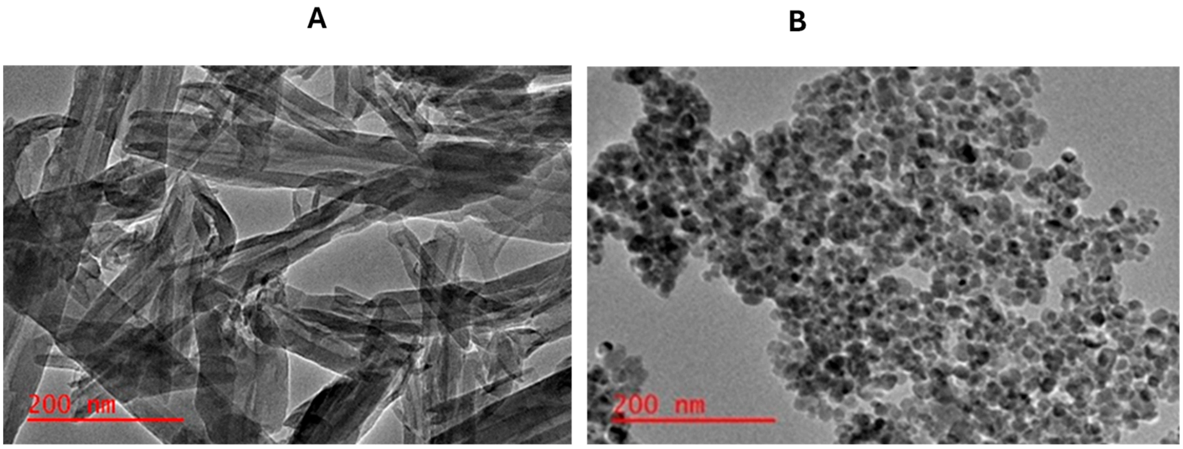

Figure S1: TEM images of (A) HNT [1] and (B) Fe_3O_4.

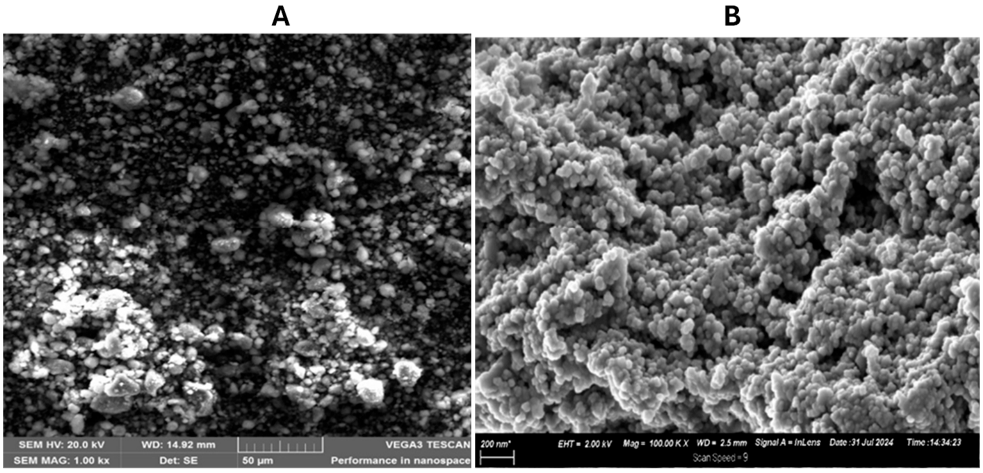

Figure S2: SEM images of (A) HNT (B) Fe_3O_4.

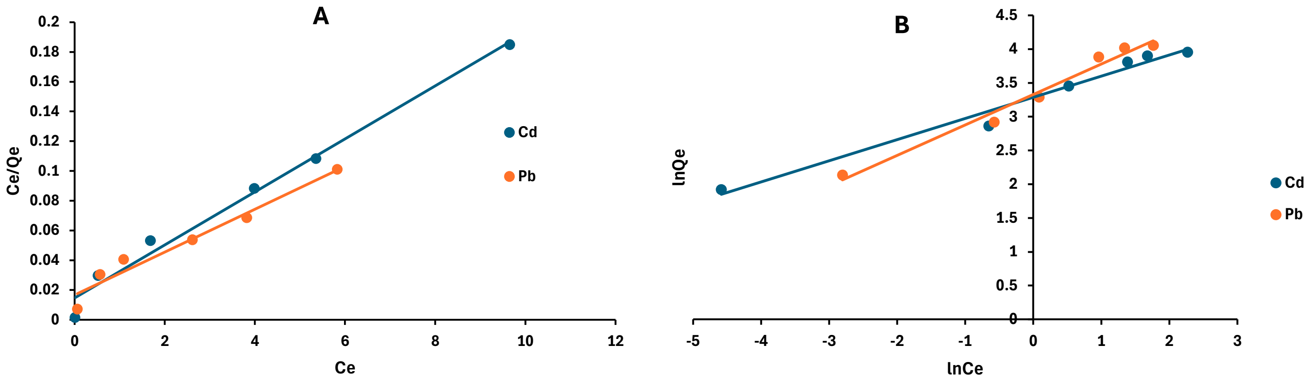

Figure S3: (A) Langmuir isotherm plot for the adsorption of Cd and Pb using Fe_3O_4@HNT-SA, (B) Freundlich isotherm plot for Cd and Pb using Fe_3O_4@HNT-SA.

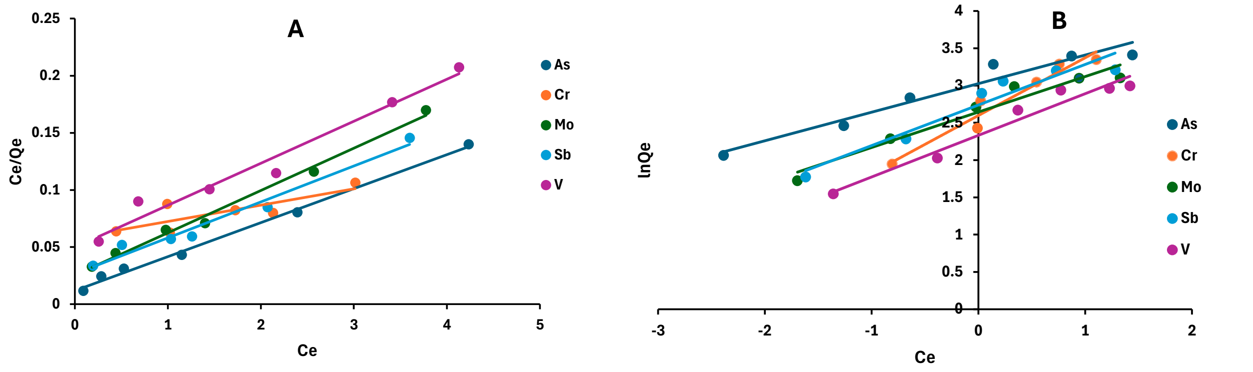

Figure S4: (A) Langmuir isotherm plot for the adsorption of As, Cr, Mo, Sb and V using Fe_3O_4@HNT-CTS (B) Freundlich isotherm plot for As, Cr, Mo, Sb and V using Fe_3O_4@HNT-CTS.

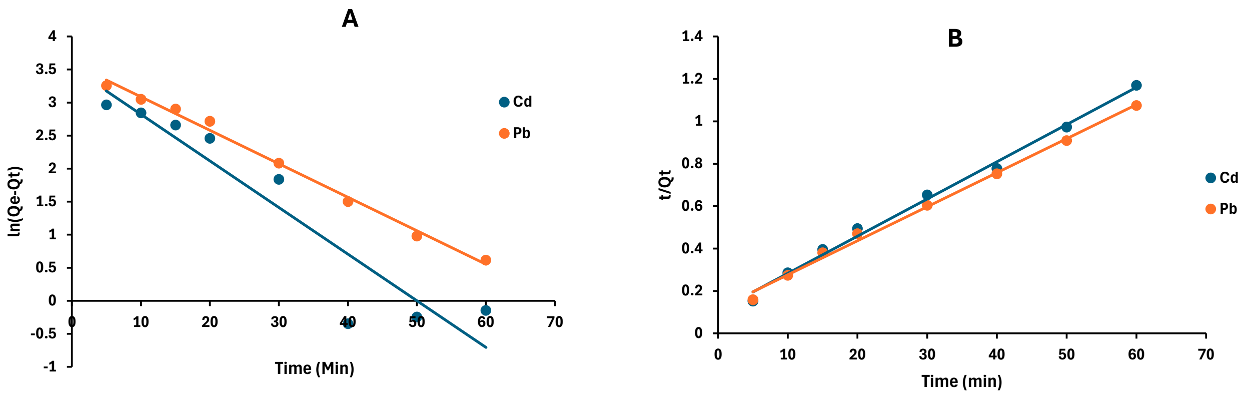

Figure S5: (A) Pseudo first order kinetics and (B) pseudo second order kinetics of Cd and Pb using Fe3O4@HNT-SA adsorbent.

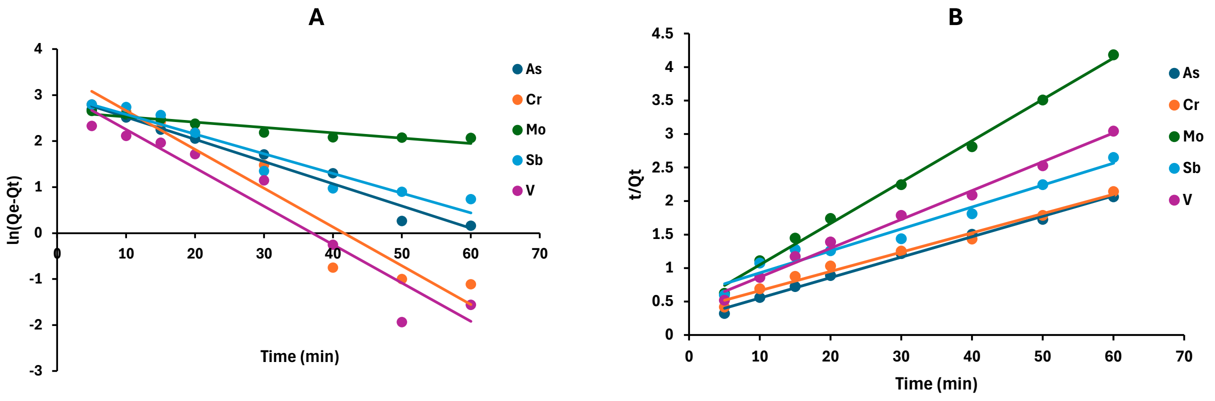

Figure S6: (A) Pseudo first order kinetics and (B) pseudo second order kinetics of As, Cr, Mo, Sb and V using Fe_3O_4@HNT-CTS.

Table S1. The effect of interfering studies on the adsorption of Cd and Pb using Fe_3O_4@HNT-SA.

Interfering ion	Interference conc (Mg/L)	%R Cd	%R Pb
**unspiked**	0	98.06	97.85
**Mg**	20	95.54	94.74
**Na**	20	97.23	97.80
**Cr**	20	95.23	92.56
**Zn**	20	84.97	86.89

Table S2. The effect of interfering studies on the adsorption of As, Cr, Mo, Sb and V onto Fe_3O_4@HNT-CTS.

Interfering ions	Interference conc (Mg/L)	%R As	%R Cr	%R Mo	%R Sb	%R V
**Unspiked**	0	77.00	85.24	86.55	65.23	100
**Cd**	20	76.57	86.63	79.32	60.67	99.09
**Fe**	20	73.55	84.57	80.78	63.88	97.22
**Na**	0	76.69	83.56	85.66	64.56	96.98
**Pb**	20	75.00	80.23	81.23	63.10	95.23
**Zn**	20	73.4	84.1	83.89	62.56	98.84
**Mg**	20	74.5	80.00	85.66	65.44	96.57

Reference

1. Mabaso, N.B.; Mnguni, M.; Nomngongo, P.N.; Nyaba, L. Ultrasonic-aided dispersive solid-phase microextraction employing magnetic halloysite nano clay for simultaneous preconcentration of lead (II) and cadmium (II). *Green Anal. Chem.* **2025**, *12*, https://doi.org/10.1016/j.greeac.2025.100219.
